# Overcoming Ambient Drift and Negative-Bias Temperature Instability in Foundry Carbon Nanotube Transistors

**DOI:** 10.1021/acsami.4c22130

**Published:** 2025-03-18

**Authors:** Andrew C. Yu, Tathagata Srimani, Max M. Shulaker

**Affiliations:** † Department of Electrical Engineering and Computer Science, Massachusetts Institute of Technology, Cambridge, Massachusetts 02139, United States; ‡ Department of Electrical Engineering, Stanford University, Stanford, California 94305, United States; § Department of Electrical and Computer Engineering, Carnegie Mellon University, Pittsburgh, Pennsylvania 15213, United States; ∥ Analog Devices, Wilmington, Massachusetts 01887, United States

**Keywords:** carbon nanotubes, carbon nanotube field-effect transistors (CNFETs), nanomaterials, reliability, NBTI, air stability, low-dimensional materials, back-end-of-line (BEOL) technologies

## Abstract

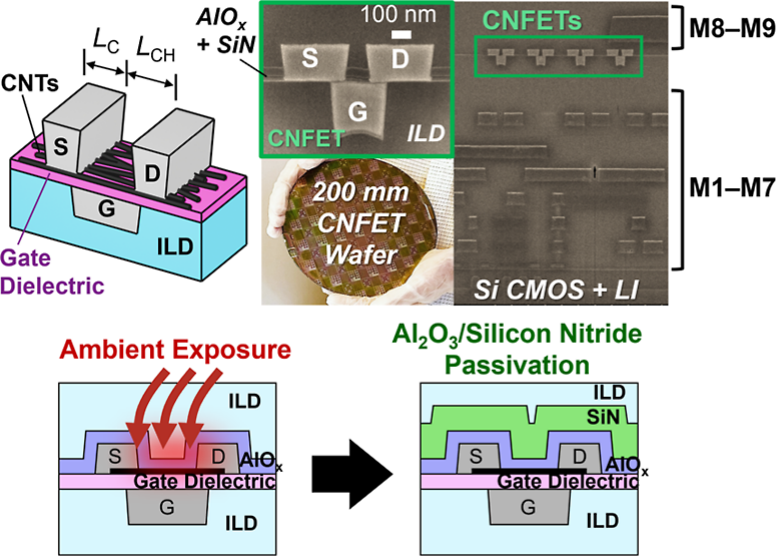

Carbon nanotube field-effect transistors (CNFETs) are promising candidates for back-end-of-line logic integration as a complementary path for continued electronic scaling. However, overcoming CNFET ambient drift (i.e., air stability) and reliability is underexplored. Here, we demonstrate that silicon nitride encapsulation limits ambient atmosphere-induced threshold voltage shift (∼8× reduction of median Δ*V*_T_ over 90 days). With stabilized nitride-encapsulated CNFETs, we characterize CNFET negative bias temperature instability (NBTI) with both DC and AC stress across the electric field, temperature, gate oxide thickness, and stress frequency. AC pulsed operation significantly improves CNFET NBTI vs DC operation across a wide frequency range of 1 kHz–10 MHz. A 20% duty cycle AC operation at 10 MHz could extend the NBTI time to failure by > 10^4^× vs DC for a target |Δ*V*_T_| tolerance ≤100 mV with a gate bias *V*_GS,Stress_ = −1.2 V at 125 °C. This work improves our understanding of overcoming ambient drift and BTI reliability in CNFETs.

## Introduction

Back-end-of-line (BEOL) integration of transistor technologies on silicon CMOS provides a complementary scaling path along the vertical dimension.^1,2^ Among the various candidates explored today^2^ (e.g., carbon nanotubes, 2D transition metal dichalcogenides, oxide semiconductors), carbon nanotube (CNT) field-effect transistors (CNFETs) show great promise: (1) CNFETs overcome major process integration challenges (e.g., through new VLSI CNT purification techniques,^3^ robust p- and n-type doping,^4^ silicon fab-compatible CNFET contact fabrication^5^) and have undergone lab-to-fab to commercial silicon CMOS foundries;^6−8^ (2) CNFET VLSI-design frameworks have been developed,^6,9^ and complex CNFET CMOS circuits, e.g., 16 bit RISC-V and SRAM arrays, have been demonstrated;^6,9,10^ and (3) CNFETs are projected to achieve significant (>7×) energy delay product benefits vs silicon FETs at extremely scaled 2 nm technology nodes.^11^ However, BEOL-compatible CNFET reliability is still not well characterized, specifically the change in electrical characteristics over time and operating conditions (e.g., bias stress, temperature, and target operating frequency). This presents a challenge to ensuring correct circuit operation over a device’s lifetime. Especially, bias stress-induced effects in threshold voltage (*V*_T_) shift within BEOL CNFET circuits could significantly alter or worsen functionality and/or throughput. Prior works have studied interface traps between CNTs and gate dielectrics and their impact on CNFET hysteresis^12−17^ but did not assess the reliability implications (e.g., bias-temperature instability, BTI, a key limiter in logic circuit lifetime). Other studies which have characterized BTI in CNFETs do not use silicon CMOS BEOL-compatible CNFET fabrication^18,19^ or use unconventional techniques to study BTI (which are therefore not used in silicon transistor BTI testing). In particular, some studies (1) focused on long-channel/unencapsulated CNFETs with thick SiO_2_ gate dielectrics, e.g., equivalent oxide thickness, EOT > 50 nm, not representative of a scaled BEOL CNFET,^18,20^ (2) used exotic gate dielectrics and silicon-foundry-incompatible processing, e.g., yttrium oxide gate dielectric, metal evaporation and lift-off, etc.,^19^ and/or (3) used BTI characterization techniques such as slow *I*_D_–*V*_GS_ that allows relaxation and underestimates BTI.^19,21^

In this work, we address both air stability and BTI reliability of CNFETs fabricated using a silicon BEOL-compatible CNFET fabrication flow on 200 mm wafers.^5,7^ We characterize and improve CNFET air stability using silicon nitride (SiN_*x*_) encapsulation. With stabilized CNFETs, we characterize DC and AC negative-bias temperature instability (NBTI) for a range of gate voltage bias, stress frequency, stress duty cycle, gate dielectric equivalent oxide thickness (EOT), and temperature using similar on-the-fly BTI testing protocols used in silicon.^22−24^ Our measurements and analyses show the following new insights.

(1) Silicon nitride (SiN_*x*_) encapsulation limits threshold voltage drift (Δ*V*_T_) in CNFETs, showing >8× improved median Δ*V*_T_ shift with SiN_*x*_ encapsulation (median Δ*V*_T_ ∼ 54 mV) vs baseline (median Δ*V*_T_ ∼ 450 mV) after 90 days in air. SiN_*x*_ encapsulation thus ensures BTI measurements are not impacted by ambient Δ*V*_T_ shift during the measurement period. NBTI-induced Δ*V*_T_ in SiN_*x*_-encapsulated CNFETs follows similar power level dependence on gate oxide electric field ξ_ox_’ and stress time as observed in silicon FETs.^25,26^
(2) Temperature does not increase the long-term magnitude of Δ*V*_T_ (e.g., > 10^3^ seconds) but causes the “peak” Δ*V*_T_ to occur sooner and significantly lengthens Δ*V*_T_ relaxation time.
(3) AC stress (i.e., pulsed gate operation) improves NBTI tolerance in SiN_*x*_-encapsulated CNFETs, e.g., > 10^4^× relaxed NBTI time to failure using AC 20% duty cycling vs DC for a target |Δ*V*_T_| tolerance ≤100 mV at gate stress bias *V*_G,Stress_ = −1.2 V and frequency = 10 MHz. We find that AC stress NBTI |Δ*V*_T_| is not impacted by AC frequencies from 1 kHz to 10 MHz, suggesting these benefits are stable across this frequency range.

This is the first comprehensive experimental analysis of DC and AC stress-induced NBTI in SiN_*x*_-stabilized CNFETs, from which we derive new insights into the CNT interface and oxide charge trapping mechanisms. We experimentally show that AC gate stress pulse cycling reduces NBTI Δ*V*_T_ accumulation, which means BEOL circuit workloads with cycled operation could have significantly, i.e., > 10^4^×, enhanced lifetime than what would be estimated from conservative DC NBTI measurements. This work improves our understanding of how to overcome ambient drift and BTI reliability in CNFETs, which are necessary for BEOL logic design and integration.

## Results and Discussion

### SiN_*x*_ Encapsulation Limits Ambient Drift

**Figure 1:**
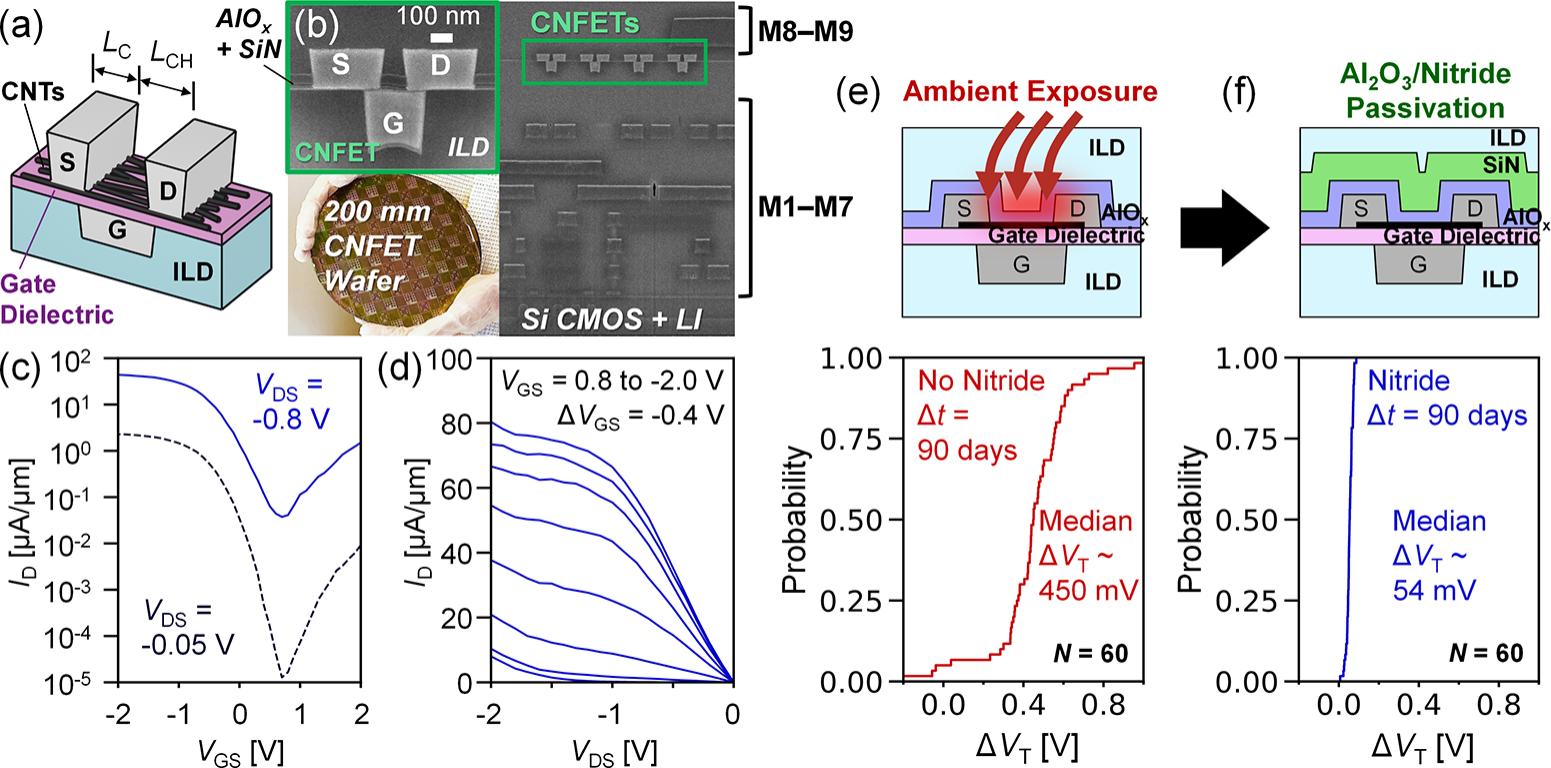
(a) Schematic of the bottom gate carbon nanotube field-effect transistor (CNFET) structure integrated in foundry back-end-of-line (BEOL) with encapsulation not shown for clarity. (b) Photo of a 200 mm silicon CMOS wafer with the CNFET integrated in BEOL between metal layers M7 and M8, shown in cross-section SEM micrographs. Zoom-in of CNFET with a gate length *L*_G_ = 260 nm, a channel length *L*_CH_ = 120 nm, and a contact length *L*_C_ = 300 nm. Typical measured (c) *I*_D_–*V*_GS_ and (d) *I*_D_–*V*_DS_ characteristics of PMOS CNFETs (*L*_CH_ = 120 nm, *L*_C_ = 160 nm, *W* = 4 μm, approximate linear CNT density of 20–50 CNTs/μm per^8^). CNFET silicon nitride passivation improves threshold voltage *V*_T_ ambient drift over time. (e) Baseline CNFET with Al_2_O_3_ + BEOL interlayer dielectric (ILD, SiO_*x*_) encapsulation (*L*_CH_ = 600 nm, *L*_C_ = 1 μm) and CDF of extracted Δ*V*_T_ shift for each CNFET after 90 days in an ambient atmosphere. (f) CNFETs with Al_2_O_3_ + SiN_*x*_ + SiO_*x*_ passivation and CDF of extracted Δ*V*_T_ shift for each SiN_*x*_ passivated CNFET after 90 days in an ambient atmosphere.

Figure 1a–b shows a schematic of our silicon BEOL-compatible CNFET structure with cross-section scanning electron micrographs of example CNFETs integrated on 200 mm wafers in the silicon CMOS BEOL from a commercial silicon foundry.^7^ Our CNFET fabrication flow^5,8^ begins with a bottom gate stack made with high-k oxide (AlO_*x*_ + HfO_*x*_) gate dielectric deposited via atomic layer deposition (ALD) on top of embedded tungsten metal gates. After that, purified 99.99% semiconducting CNTs are deposited on top, and O_2_ plasma is used to remove CNTs outside of the transistor channel regions. Source/drain contacts are fabricated either using evaporated metal + lift-off^6,8^ or using lift-off-free etch/fill/planarization with a sputtered metal liner + tungsten fill via chemical vapor deposition (CVD).^5,7,27^Figure 1c–d presents typical transistor *I*_D_–*V*_GS_ and *I*_D_–*V*_DS_ electrical characteristics for lift-off-free CNFET PMOS fabricated using an ALD TiN + sputtered W metal liner which is used in this work to study NBTI. Details on transistor electrical characterization are in Methods.

A prior challenge in CNFETs is threshold voltage shift (Δ*V*_T_) over time from atmospheric adsorbed water doping,^12,14,16^ termed “ambient drift”. We find that SiN_*x*_ is key to passivating foundry wafer-scale CNFETs from an ambient atmosphere.^18^ Only Al_2_O_3_ + SiO_*x*_ encapsulation shows CNFET Δ*V*_T_ shift ∼ 450 mV after 90 days of exposure to the atmosphere, whereas Al_2_O_3_ + SiN_*x*_ + SiO_*x*_ encapsulation limits Δ*V*_T_ shift to ∼54 mV, a ∼8× improvement (Figure 1e–f). Additional details on SiN_*x*_ encapsulation and *I*_D_–*V*_GS_ characteristics used to extract ambient Δ*V*_T_ shifts are in Section S1 of the Supporting Information. Thus, our CNT channels in Figure 1a–d are encapsulated with 20 nm Al_2_O_3_ deposited via ALD at 200 °C followed by plasma-enhanced chemical vapor deposition (PECVD) of 50 nm BEOL silicon nitride (SiN_*x*_) deposited at 400 °C and 200 nm BEOL dielectric (SiO_*x*_) deposited at 350 °C. SiN_*x*_-encapsulated ambient stabilized CNFETs are used in all the following sections to characterize bias temperature instability, BTI, which ensures that the measured Δ*V*_T_ shift is due to BTI and not ambient drift over the course of measurement.

## DC NBTI

**Figure 2:**
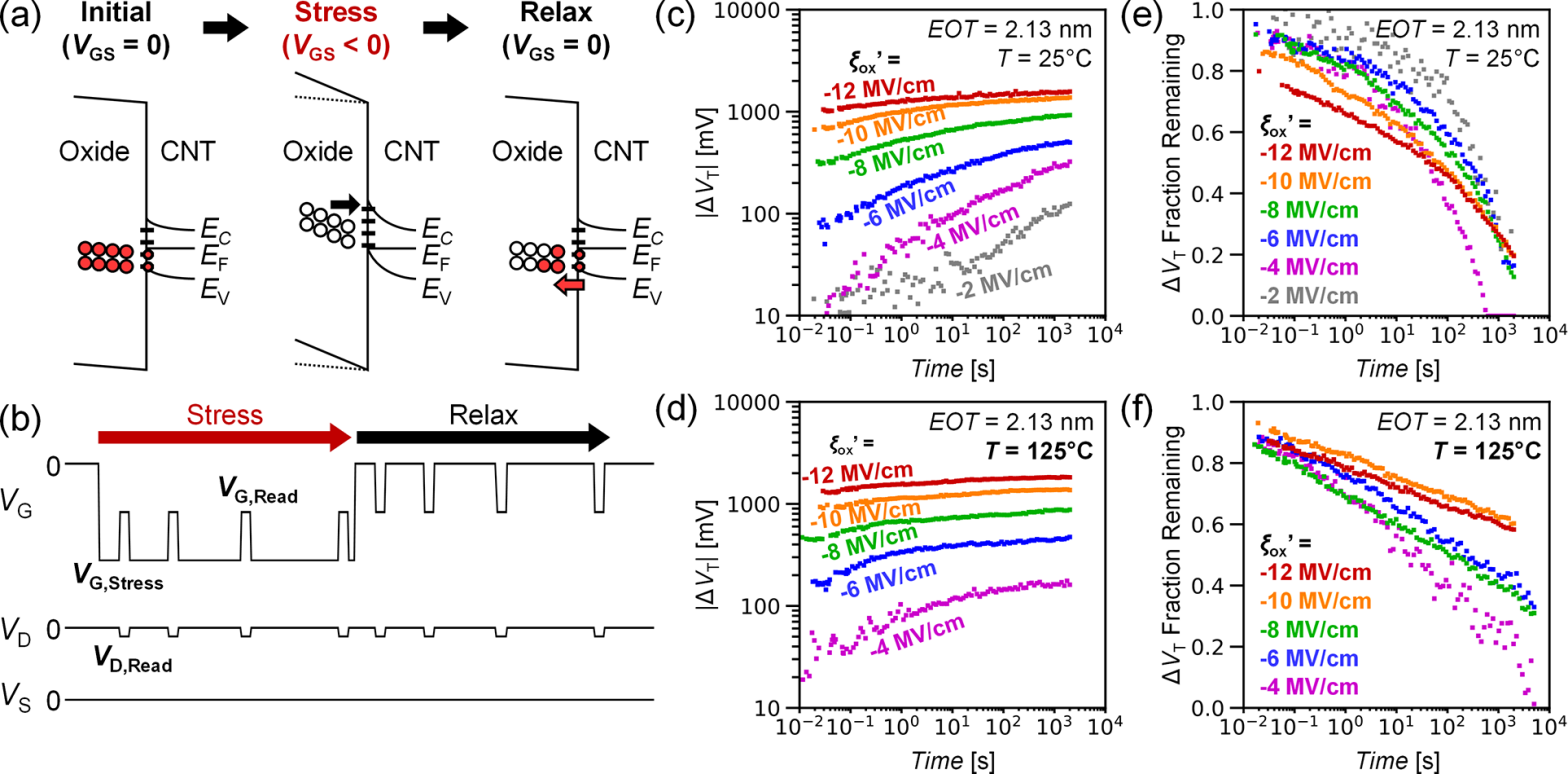
(a) Qualitative band diagrams showing speculated cause of NBTI from charge trapping and detrapping at the CNT–oxide interface and oxide bulk traps, based on conventional silicon FET understanding.^28,29^ Red circles represent electron-filled trap states, and white circles represent empty trap states. (b) DC NBTI stress/relaxation on-the-fly characterization waveforms. DC NBTI in CNFETs with ∼70 Å gate oxide and measured EOT ≈ 2.13 nm (effective relative dielectric constant of 12.8). (c) Room-temperature and (d) 125 °C DC NBTI vs stress time at different oxide electric fields. Relative Δ*V*_T_ recovery as a fraction of final stress Δ*V*_T_ vs relaxation time at (e) room temperature and (f) 125 °C. Each stress and relaxation versus time curve at each electric field value comes from the same measurement.

A key consideration of this work is to understand the change in CNFET electrical characteristics during operation under various conditions (gate stress, temperature, frequency, etc.) to guide logic design. Negative gate bias used to operate CNFET PMOS induces negative threshold voltage shift Δ*V*_T_, i.e., negative bias temperature instability, NBTI, which is observed in silicon PMOS.^28^ BTI-induced Δ*V*_T_ is a key reliability challenge in CMOS circuits and must be limited to ensure the correct circuit functionality over a lifetime of operation. BTI-induced Δ*V*_T_ is the physical origin of hysteresis commonly observed in CNFET *I*_D_–*V*_GS_ measurements, which occurs in both unencapsulated^12,14,15^ and dielectric encapsulated CNFETs.^17,18^ The physical origin of NBTI in our fully dielectric encapsulated CNFET PMOS is speculated to be from oxide charge trapping (qualitative diagram in Figure 2a, additional discussion on our CNT-oxide interface is in Section S2 of the Supporting Information). Filling of oxide states may be facilitated by trap-assisted tunneling from interface traps at the CNT–oxide interface.^29^ The CNFET density of interfacial traps *D*_it_ in this work is estimated to be *D*_it_ ∼10^13^ cm^–2^ eV^–1^ from *I*_D_–*V*_GS_ characteristics (details in Section S3 of the Supporting Information), similar to prior published CNFETs^15,30^ and ∼ 1000× higher than a typical hydrogen-passivated Si/SiO_2_ interface in conventional silicon MOSFETs.^28,31,32^

**Figure 3:**
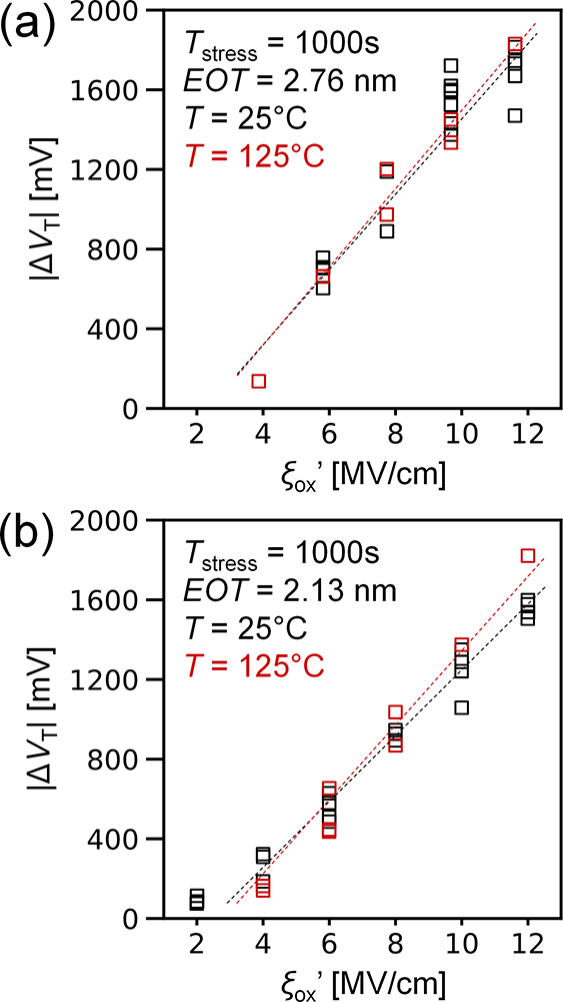
DC NBTI in CNFETs vs gate oxide electric field ξ_ox_’ at room temperature 25 and 125 °C nm sampled after 10^3^ seconds of stress for (a) *EOT* ≈ 2.76 nm (effective relative dielectric constant of 14.6) and (b) EOT ≈ 2.13 nm (effective relative dielectric constant of 12.8).

We extract NBTI Δ*V*_T_ vs time by applying a DC constant gate stress and log-time spaced sampling the change in drain current with spot on-the-fly current measurements (Figure 2b, similar to conventional silicon BTI testing, with measured controller limited read pulse width ∼1 and ∼ 1 ms delay from stress to relaxation which is accounted for in relaxation times). Characterization of the DC NBTI measurement setup is in Section S4 of the Supporting Information. Figure 2c–f shows the DC NBTI threshold shift Δ*V*_T_ vs stress and relaxation time characterized for different parameters which typically affect the rate and magnitude of NBTI:^25,28^(1) oxide electric field normal to the channel (from gate bias), normalized as ξ_ox_’ = (*V*_GS_–*V*_T_)/EOT, where *V*_T_ is the prestress threshold voltage,^25,33,34^ (2) temperature, and (3) equivalent oxide thickness, EOT. Figure 3 shows DC NBTI Δ*V*_T_ sampled at a 10^3^ s stress time vs normalized oxide electric field sampled across multiple CNFETs. Typical NBTI models characterize Δ*V*_T_ with a power law dependence versus oxide electric field and time at a given temperature and EOT^25,26^
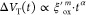
1where *m* and α are the empirical fitting parameters. From Figures 2 and 3, we observe the following: (1) the magnitude of NBTI-induced Δ*V*_T_ appears to be linearly dependent on the normalized oxide electric field for |ξ_ox_’| > 2 MV/cm; (2) temperature does not appear to increase the long-term magnitude of Δ*V*_T_ (e.g., > 10^3^ seconds); (3) however, higher temperature causes the “peak” Δ*V*_T_ to occur sooner and lengthens the Δ*V*_T_ relaxation time. Additional fitting and analysis of CNFET Δ*V*_T_ relaxation in comparison to the silicon FET “universal relaxation” model^35,36^ is in Section S5 of the Supporting Information

## AC/Pulsed NBTI

**Figure 4:**
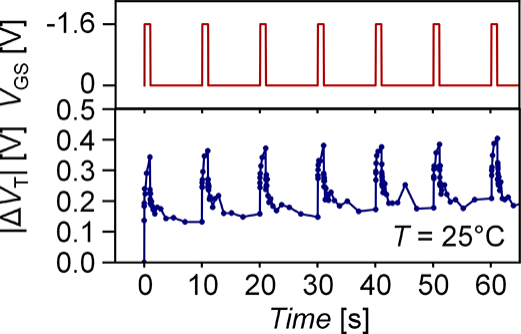
Measured CNFET Δ*V*_T_ with pulsed-gate stress–relax cycles, which slows buildup of BTI-induced Δ*V*_T_.

**Figure 5:**
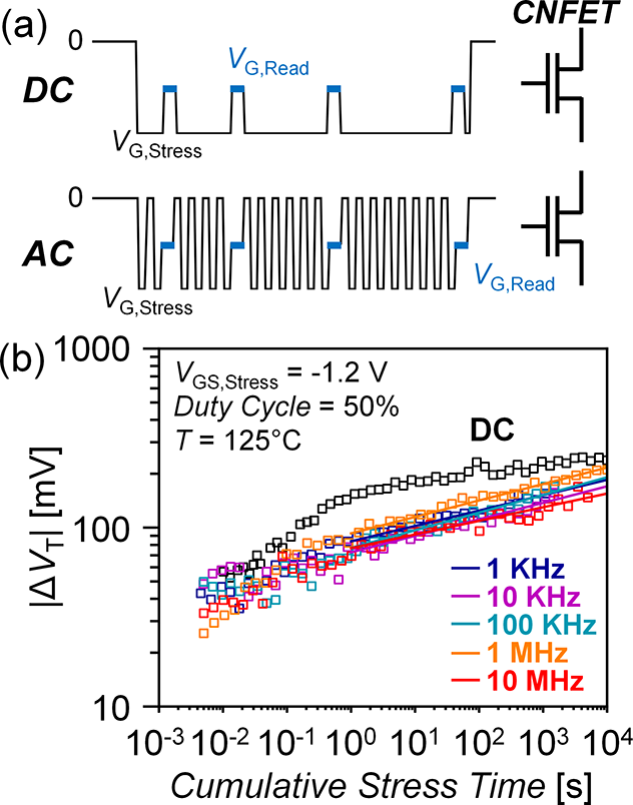
(a) DC vs AC NBTI stress waveforms using a relax–stress–measure AC stress pattern. The controller read pulse width is 1–2 ms. (b) AC NBTI at 125 °C, EOT ≈2.13 nm, *V*_GS,stress_ = −1.2 V) across a range of frequencies from 1 kHz to 10 MHz and *D* = 50% duty cycle, showing a lack of AC NBTI frequency dependence. Cumulative stress time sums only time when stress is applied and does not include relaxation time.

For typical logic applications, CNFET gate bias will not be a constant DC value but rather will be pulsed on and off. This allows time for CNFETs to relax between bias stress, which reduces the long-term buildup of NBTI Δ*V*_T_.^37^Figure 4 shows an example of measured CNFET *V*_T_ over time with pulsed stress–relax cycles, showing how periodic relaxation cycles slow the buildup of BTI-induced Δ*V*_T_. This behavior is formalized and characterized using an AC NBTI stress pattern with frequency f with duty cycle D, which is the fraction of each cycle that the stress voltage is applied (qualitative characterization waveforms in Figure 5a). We apply a relax–stress–measure AC NBTI pattern which is expected to better capture both deep oxide and shallow interface traps.^37^Figure 5b shows reduced AC NBTI Δ*V*_T_ shift over cumulative stress time (summing only time when stress is applied, not any relaxation time) with AC stress vs DC at 125 °C across frequencies (1 kHz to 10 MHz, duty cycle *D* = 50%). This benefit occurs even with a longer relaxation time at elevated temperatures. Additional AC NBTI characterization at room temperature 25 °C and with different stress–relax and relax–stress patterns^37^ is in Section S6 of the Supporting Information.

**Figure 6:**
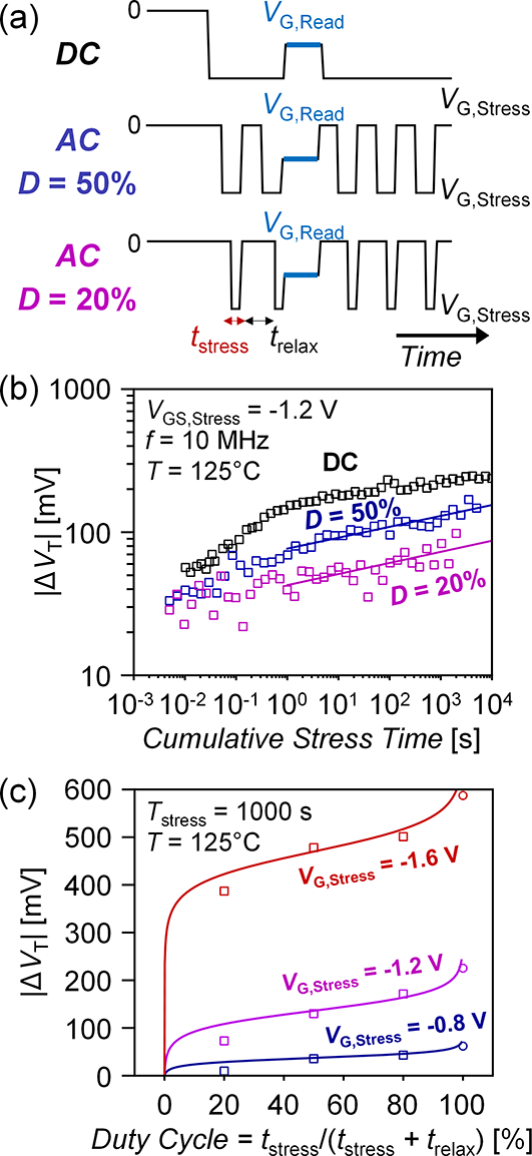
(a) DC versus pulsed AC gate voltage stress waveforms at different duty cycles *D*. (b) CNFET DC vs AC NBTI at 125 °C using 10 MHz AC stress with duty cycles *D* = 50% and D = 20% at *V*_GS,stress_ = −1.2 V. CNFETs have EOT ≈ 2.13 nm. (c) CNFET AC NBTI vs duty cycle at different stress biases. Open points are measurements at 125 °C, a 10 MHz stress, after a total accumulated stress time *T*_stress_ = 10^3^ s (CNFETs have *EOT* ≈ 2.13 nm). Solid lines fitted using the model described in refs (29 and 37).

Reducing the AC stress duty cycle *D* is expected to reduce accumulated NBTI Δ*V*_T_ by providing more time for charge relaxation (Figure 6a). Figure 6b shows DC versus AC NBTI Δ*V*_T_ measured with 10 MHz stress duty cycle *D* = 50% and *D* = 20%. We observe clear reduction in Δ*V*_T_ with lower AC stress duty cycles at cumulative stress times >1 s. Figure 6c shows the plotted trend in Δ*V*_T_ sampled at total cumulative stress time *T*_stress_ = 1000 s for different duty cycles and gate bias (the measured AC NBTI vs time data for each gate bias is in Section S7 of the Supporting Information). Our measured behavior in Figure 6c is fitted to a model for silicon AC NBTI based on interface trap tunneling to and from oxide states, originally developed by Tewksbury (1992)^29,37^
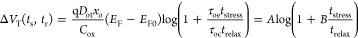
2
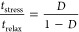
3where *D*_ot_ is the trap density in the oxide, *x*_0_ is a characteristic tunneling depth, *C*_ox_ is the gate dielectric capacitance, *E*_F_ – *E*_F0_ is the energy range of traps, τ_oc_ and τ_oe_ are the trap capture and emission time constants, and *t*_stress_ and *t*_relax_ are the stress and relax time portions of the periodic AC stress waveform, respectively (labeled in Figure 6a). For model fitting, the trap dynamics are simplified into two empirical fitting parameters, *A* and *B*. The general shape of this silicon charge trapping model fits the measured duty cycle dependence of Δ*V*_T_ after a fixed cumulative stress time *T*_stress_ in our measured CNFETs undergoing AC NBTI stress. This supports the interface and oxide charge trapping mechanisms proposed in Figure 2a as the origin of CNFET NBTI effects.

To project how AC duty cycling operation improves NBTI reliability, Figure 6b can be used to project a “NBTI time-to-failure”. For example, if a circuit specification is that the maximum tolerable |Δ*V*_T_| is < 100 mV, the “NBTI time-to-failure” is the cumulative stress time when the Δ*V*_T_ exceeds this threshold. Under conditions in Figure 6b and DC stress, this occurs at *T*_stress_ ∼ 10^–1^ seconds, while under AC stress with *D* = 50%, this time to failure is extended out by > 10^2^× relative to DC, and with *D* = 20%, it is > 10^4^× relative to DC. We illustrate this analysis in Section S8 of the Supporting Information. This represents a significant NBTI tolerance enhancement under pulsed AC operation versus a DC operation. This shows a methodology to operate CNFETs in pulsed logic workloads with sufficient duty cycling that can overcome NBTI.

## Conclusions

In conclusion, this work advances our understanding and ability to stabilize CNFET electrical characteristics in an ambient atmosphere and during operation. We experimentally demonstrate a method to limit foundry CNFET ambient atmosphere-induced threshold voltage Δ*V*_T_ shift via silicon nitride encapsulation. This enables us to analyze DC and AC NBTI with a negligible influence of ambient induced Δ*V*_T_. We characterize DC and AC pulsed CNFET NBTI at various operating conditions (gate stress, temperature, frequency, duty cycle). We show that DC NBTI is conservative, and AC operation significantly limits NBTI-induced Δ*V*_T_ accumulation at a wide range of gate voltage bias and AC stress frequency. These results and techniques enable future work to develop more accurate models for circuit designers to codesign appropriate circuit-level tolerances and mitigations for CNFET behavior shift.

## Methods

CNFETs in this work are fabricated in a silicon CMOS foundry on 200 mm wafers by using conventional front-end-of-line silicon tooling. Details on CNFET process flow are described in refs (5, 7, and 8). CNFET *I*_D_–*V*_GS_ and *I*_D_–*V*_DS_ electrical measurements are performed in air at room temperature in a Cascade Summit 12000 probe station with a Keysight B1500A semiconductor parameter analyzer. Room-temperature BTI stress characterization is performed in a Cascade Summit 12000 probe station in ambient atmospheric conditions. Elevated temperature stress is performed in a Cascade probe station with a heated chuck in an atmosphere with nitrogen flowing over the wafer. Pulsed on-the-fly BTI measurements are implemented using a National Instruments chassis with a PXIE 6570 pulse driver interfaced with Python control scripts. Characterization of the DC and AC NBTI measurement setup is in Sections S4–S6 of the Supporting Information. EOT is characterized from the measured C–V characteristics of metal–insulator–metal capacitors of the gate dielectric on the same dies on the same wafer next to the CNFETs characterized for BTI.

## Supporting Information Available

CNFET electrical measurements with/without silicon nitride, CNT–oxide interface, CNFET density of interfacial trap estimation, DC NBTI setup characterization, CNFET relaxation, CNFET AC NBTI setup and additional characterization, CNFET AC NBTI additional duty cycle characterization, and illustration of AC NBTI time-to-failure improvement vs DC (PDF)
