# Supplementary material for: Overcoming Ambient Drift and Negative-Bias Temperature Instability in Foundry Carbon Nanotube Transistors
Source: ACS Appl Mater Interfaces. 2025 Mar 18;17(13):20411–7. doi: 10.1021/acsami.4c22130 (PMC11969425; doi:10.1021/acsami.4c22130)
Supplement: Supplementary file 1 — am4c22130_si_001.pdf [file am4c22130_si_001.pdf]

Supporting Information: Overcoming Ambient Drift and Negative-Bias Temperature Instability in Foundry Carbon Nanotube Transistors

Andrew C. Yu[1], Tathagata Srimani[2,3,*], Max M. Shulaker[1,4]

[1] *Department of Electrical Engineering and Computer Science, Massachusetts Institute of Technology, Cambridge, MA 02139, USA*

[2] *Department of Electrical Engineering, Stanford University, Stanford, CA 94305, USA*

[3] *Department of Electrical and Computer Engineering, Carnegie Mellon University, Pittsburgh, PA* 15213*, USA*

[4] *Analog Devices, Wilmington, MA 01887, USA*

*Corresponding author, contact: tsrimani@andrew.cmu.edu

S1. CNFET SiN_x Encapsulation Characterization Details

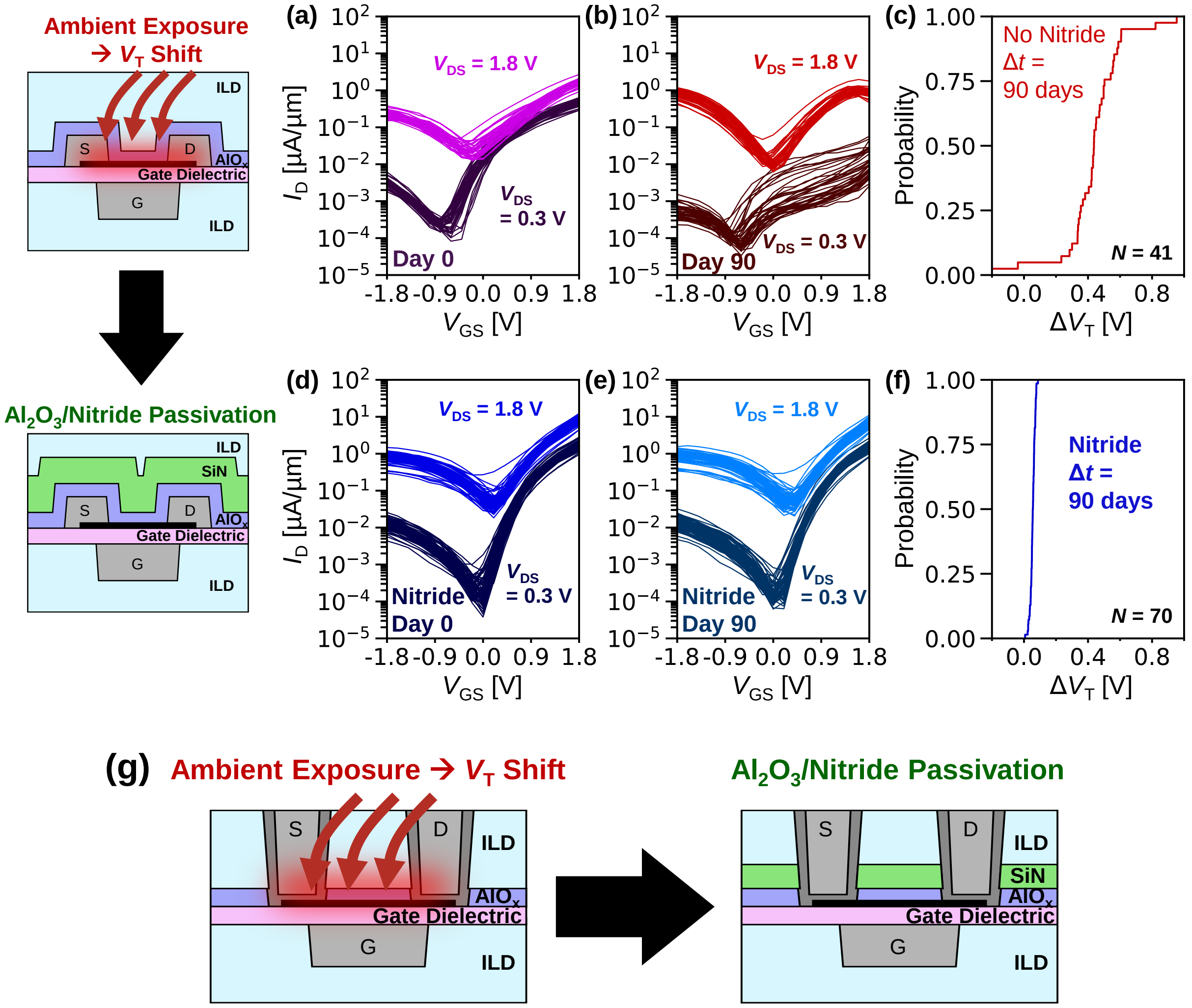

Figure S1. CNFET channel nitride passivation to improve ambient drift. Baseline foundry CNFET with lift-off Ti contacts and Al_2O_3 + ILD encapsulation (L_{CH} = 600 nm, $L_{CONTACT}$ = 1 μm): (a) Overlay of I_D-V_{GS} measured <1 day after receiving, (b) same I_D-V_{GS} measured 90 days after receiving, (c) CDF of extracted ΔV_T shift for each CNFET after 90 days in ambient atmosphere (N = 41). CNFET with lift-off Ti contacts with Al_2O_3 + nitride + ILD passivation: (d) Overlay of I_D-V_{GS} measured <1 day after receiving, (e) same I_D-V_{GS} measured 90 days after receiving, (f) CDF of extracted ΔV_T shift for each CNFET after 90 days in ambient atmosphere (N = 70). (g) Incorporation of Al_2O_3 + nitride passivation into lift-off-free CNFET channel stack.

A challenge in CNTs is threshold voltage shift and hysteresis from atmospheric adsorbed water doping [1–3]. Lau (2019) found SiN encapsulation is sufficient to passivate CNFETs to significantly reduce ambient threshold voltage drift. Unencapsulated CNFETs drift by >100% of V_{DD} over 8 days in ambient air, while SiN encapsulated CNFETs drift by <1.7% of V_{DD} over 56 days in

ambient air [4]. Nitride passivation is transferred and adopted into foundry CNFETs (**Figure S1**). Baseline foundry CNFET NMOS with lift-off Ti contacts using only Al_2O_3 + ILD encapsulation show median ambient induced threshold shift ΔV_T ~ 437 mV (~24% of supply V_{DD} = 1.8 V) and visually degraded I_D-V_{GS} characteristics after 90 days exposure to atmosphere (L_{CH} = 600 nm, $L_{CONTACT}$ = 1 μm, N = 41 CNFETs, **Figure S1a-c**). Incorporating ~100 nm of nitride after the Al_2O_3 passivates the CNT channel and blocks ambient threshold voltage drift over time. Passivated foundry CNFET NMOS with lift-off Ti contacts and Al_2O_3 + nitride + ILD encapsulation now show stable I_D-V_{GS} characteristics after 90 days exposure to ambient atmosphere, with median ambient induced threshold shift ΔV_T ~ 55 mV (~3% of supply V_{DD} = 1.8 V), a ~7.9× reduction in ambient induced ΔV_T from baseline (N = 70 CNFETs, **Figure S1d-f**). V_T is extracted using the method of linearly extrapolating the slope of the linear regime I_D-V_{GS} characteristics to the x-intercept from the point of max $g_m = dI_D/dV_{GS}$ at low drain bias (V_{DS} = 0.3 V is used). ΔV_T is taken as the difference in V_T extracted at day 0 and after day 90.

Figure S1g shows the nitride passivation seamlessly incorporated into the lift-off-free CNFET process flow with no changes to the source/drain contact via etch (a single plasma etch is used for both nitride + ILD). This combined Al_2O_3 + nitride passivation in all CNFETs measured in this work allows ambient induced ΔV_T to have negligible impact on our measured results for bias temperature instability.

S2. CNT-Gate-Oxide Interface and Origins of BTI

Hysteresis in CNFET electrical measurements is well known phenomena [1–3] and is consistent with bias temperature instability (BTI) caused by interface and oxide traps which is a well-known challenge in silicon MOSFETs. The source of hysteresis is hypothesized to be from bias temperature instability (BTI) [5,6]. For our PMOS CNFETs, we specifically characterize negative bias temperature instability (NBTI) because these are operated with negative gate biases. The dynamics of BTI involve the bonding and trapping physics at the semiconductor-insulator interface. **Figure S2** compares a typical hydrogen passivated Si-SiO_2 interface versus a hypothesized CNT-high-K oxide interface.

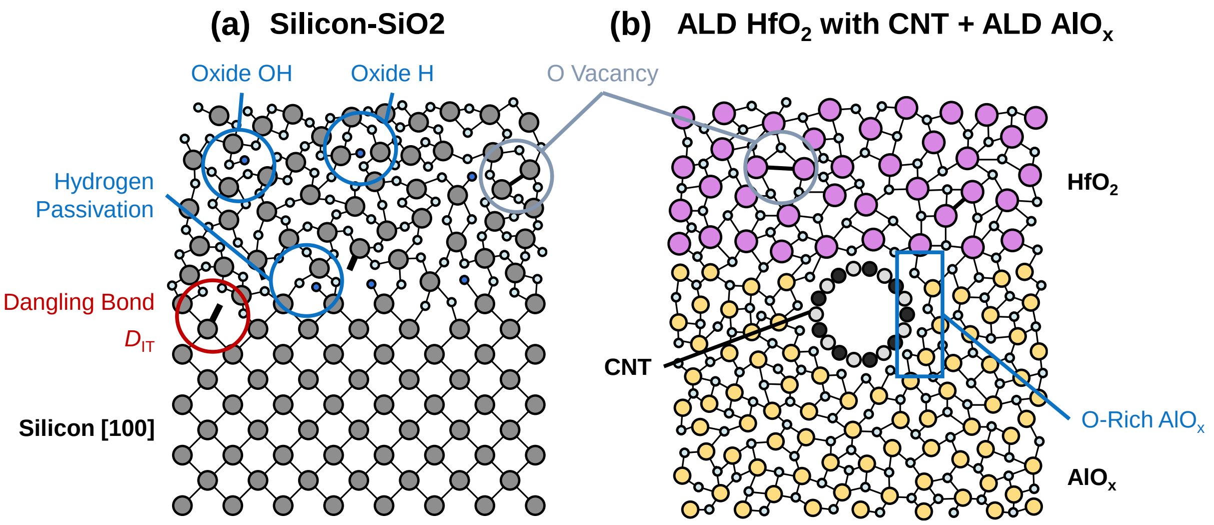

Figure S2. Hypothetical CNT-oxide interface. (a) Cartoon of Si-SiO_2 interface with typical interface and oxide defects, drawing based on ideas in [7]. (b) Speculative cartoon of our CNT-oxide material system: ALD HfO_2 with deposited CNT plus ALD AlO_x encapsulation. An oxygen-rich AlO_x layer near the CNT is based on [8]. Note the CNT-oxide picture is depicted upside down versus our real CNFETs, in order to match the Si-SiO_2 picture. The CNT-oxide material system defects and occurrence of other chemical species like H or OH is expected, but the concentration is unknown. The source of D_{it} in the CNT-oxide interface is unknown.

In silicon PMOS, the classic understanding of silicon NBTI dynamics is based on the reaction-diffusion model (RD) involving breaking passivated Si-H bonds at the Si-SiO_2 interface during negative voltage bias and the subsequent H diffusion into the oxide [5]. Hydrogen in a forming gas (H_2/N2) anneal is well-known to passivate dangling bonds in silicon that cause surface trap states. This was reported as early as 1965 independently by Balk and Kooi [9]. A typical forming gas anneal is 10%/90% H_2/N_2 at ~400-450°C for 10-30 minutes to hydrogen-passivate dangling

silicon bonds at the Si-SiO_2 interface, which significantly reduces the density of interface traps (D_{it}) from post-oxidation/pre-anneal ~10^{12} cm^{-2} eV^{-1} to post-anneal ~10^{10} cm^{-2} eV^{-1} [5,9,10]. A cartoon of a Si-SiO_2 interface that has undergone hydrogen passivation is depicted in **Figure S2a** [7]. Remaining dangling Si bonds are the source of D_{it}. The RD model of NBTI stress and recovery in silicon is as follows: (1) negative voltage bias breaks the Si-H bonds, which generates D_{it}, (2) the positive H diffuses into the oxide generating positive fixed charge that shifts threshold voltage more negative, and (3) the "recovery" or "relaxation" occurs when bias is removed and H diffuses back to re-passivate the Si dangling bonds at the Si-SiO_2 interface. The impact of NBTI on silicon PMOS device performance is a negative threshold voltage shift due to the fixed positive charge in the oxide from the dissociated hydrogen, as well as subthreshold swing and mobility degradation from generating surface states (D_{it} increases during NBTI stress) [5]. Permanent D_{it} (and hence permanent V_T shift) is generated if H is lost and never re-passivates the silicon dangling Si bonds. Silicon NBTI is often modelled with a recoverable component and smaller but "permanent" component [11–13]. The "permanence" of NBTI in silicon is still being modelled [12,13]. The alternative theory of NBTI is hole trapping in the oxide, which is the same underlying dynamics involved in NMOS PBTI [6,14].

The characteristics of our CNT-Al_2O_3 and CNT-HfO_2 interfaces are not yet fully understood. A speculative cartoon of our CNT-oxide interfaces is depicted in **Figure S2b**. The HfO_2 is first ALD deposited prior to CNT + ALD Al_2O_3. This picture assumes amorphous HfO_2 material with average Hf-O bond coordination number of 5-7 and O-Hf bond coordination number of ~3 [15]. The ALD Al_2O_3 is depicted to be oxygen-rich near the CNT interface based on *in operando* high-energy x-ray diffraction analysis by Young et al. of ALD Al_2O_3 growth on CNTs [8]. Young et al. determine that near the CNT interface the ALD Al_2O_3 has an O/Al ratio of ~1.9, an average Al-O coordination number of ~4.4 and an O-Al coordination number of ~2.6. This trends towards an O/Al ratio of ~1.5, Al-O coordination number ~4.65 and O-Al coordination number ~2.95 after 50 ALD cycles away from the CNT, which are more in line with expected bulk amorphous θ-Al_2O_3 properties [8].

For NBTI in our CNT-oxide material system, it is unknown if the mechanics of the silicon RD model apply because the CNTs themselves do not have dangling bonds. We do not use any forming gas anneal, so the presence and/or concentration of H or OH in our oxide is unknown. From the picture in **Figure S2b**, despite the appearance of no CNT dangling bonds, the high level

of D_{it} in CNTs and high NBTI V_T shift relative to silicon suggests that the interface is not passivated and there is substantial interaction with the interfacial trap states. A hypothesis is that oxide dangling bonds at the interface could be the source of trap states in the CNT. The source of NBTI would be more in line with the charge trapping hypothesis. However, note this picture also does not include potential photoresist contamination or polymer wrapping from the semiconducting CNT sorting process, which could be additional sources of trapping [16,17]. Significant future studies outside the scope of this work will be needed to characterize and understand the bonding and trapping dynamics of the CNT-oxide interface.

S3. Estimation of CNFET Density of Interfacial Traps

While CNTs themselves do not have dangling bonds, it is not yet fully understood what terminates the oxide bonds or how the CNT carbon atoms interact with the surrounding oxide atoms. A conservative estimate of D_{it} in our CNFETs is calculated from subthreshold swing SS [18],

$$D_{\mathrm{it}} \approx \frac{C_{ox}}{q^2}\left(\frac{SS}{\ln(10)\, k_B T/\, q} - 1\right) \tag{S1}$$

where C_{ox} is the gate dielectric capacitance, SS is the subthreshold swing, q is the elementary charge, k_B is the Boltzmann constant, and T is temperature. This gives a conservative overestimate of D_{it} by lumping depletion capacitance into $C_{it} = q^2 D_{it}$ and by assuming that quantum capacitance C_q is negligible in depletion. Because subthreshold swing SS increases with CNFET channel width due to CNT variation [3], we plot SS versus channel width and estimate the value that SS approaches as channel width goes to zero. The assumption is that at large channel widths, CNT variation dominates SS, while at short channel widths approaching zero, the remaining SS is dominated by D_{it}. From plots of SS versus channel width in **Figure S3**, SS saturates for channel widths <200 nm, so an average of median SS for widths <200 nm is used to estimate D_{it}. The table in **Figure S3c** shows the calculated D_{it} for two wafers representative of CNFETs in this work ranges from 1.19×10^{13} to 1.45×10^{13} cm^{-2} eV^{-1}. Our estimated D_{it} is consistent with 2×10^{12} to 3×10^{13} cm^{-2} eV^{-1} range reported in literature in other CNFETs [19,20].

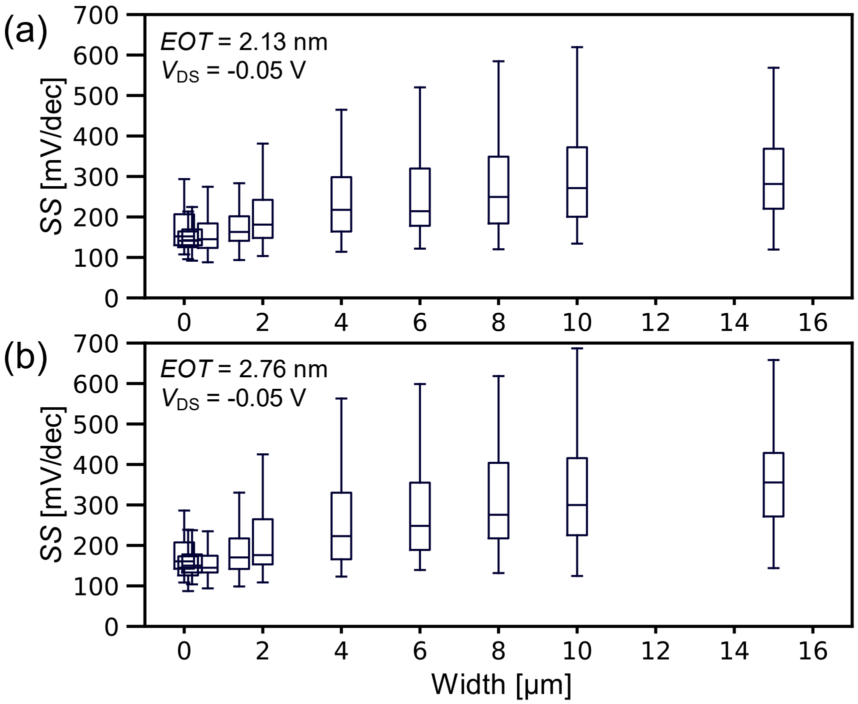

(c) Wafer	*EOT* [nm]	C_{ox} [F/cm²]	*SS* at Min. Width [mV/dec]	Estimated D_{IT} [cm^{-2} eV^{-1}]
W1	2.13	1.62×10^{-6}	145	1.45×10^{13}
W2	2.76	1.25×10^{-6}	150	1.19×10^{13}

Figure S3. Estimated CNFET D_{it} from subthreshold swing. Subthreshold swing (*SS*) versus channel width at L_{CH} = 160 nm, $L_{CONTACT}$ = 200 nm for two wafers characterized for NBTI: (a) wafer #1 (W1) with *EOT* ≈ 2.13 nm, (b) wafer #2 (W2) with *EOT* ≈ 2.76 nm. (c) Table with wafer *EOT*, measured gate dielectric capacitance C_{ox} from metal-insulator-metal capacitors on die, extracted median subthreshold swing *SS* at short <200 nm channel widths at low bias V_{DS} = -0.05 V, and estimated D_{it} from subthreshold swing assuming all degradation in *SS* from its ideal value at room temperature is due to C_{it}.

The high D_{it} in CNFETs (~10^3× greater than silicon) suggests that the CNT-oxide interface is not passivated. The working hypothesis is that these unterminated oxide bonds are a source of interfacial traps. Unlike silicon, post-gate-oxide CNFETs do not appear to see benefits from forming gas annealing. Lau et al. observed that both forming gas and nitrogen (N_2) annealing cause similar changes in subthreshold swing and V_T variation distributions post-anneal, with pure N_2 annealing providing better improvements [4]. The optimal improvement occurs with N_2 anneal

around 300°C, while both forming gas or N_2 anneal at >300°C has less improvement and eventually begins to degrade device performance [4]. Similarly, in MoS_2 which is a layered Van der Waal semiconductor that also has no dangling bonds, post-gate-oxide forming gas anneals have inconsistent effects and have also been reported to degrade device performance when annealed at >400°C [21–23].

S4. DC NBTI Measurement Details

NBTI is characterized using a pulsed on-the-fly technique with single spot drain current measurement sampling during stress illustrated by **Figure S4** [4,24]:

1. Take an initial pulsed I_D-V_{GS} curve whose max V_{GS} is less than the stress voltage $V_{GS,stress}$, with sufficient relaxation between each V_{GS} pulse to minimize induced NBTI.
2. Apply a DC or AC stress bias $V_{GS,stress}$ on the gate while holding the source/drain at zero bias.
3. Sample at log time spaced intervals at a constant voltage $V_{GS,read}$ and drain bias $V_{DS,read}$ and read a single spot drain current measurement $I_{D,read}$. The $V_{GS,read}$ is a voltage within the initial pulsed I_D-V_{GS} curve measured.
4. Interpolate the sampled current $I_{D,read}$ at time t to its equivalent gate voltage on the initial I_D-V_{GS} as V_{GS}(t). The threshold voltage shift at time t is interpreted as $\Delta V_T(t) = V_{GS}(t) - V_{GS,read}$. The simplifying assumption is that the entire I_D-V_{GS} shifts with NBTI from charge in the gate dielectric and that there is no g_m degradation. These conditions are satisfied in CNFETs measured in this work.
5. To sample relaxation, the same on-the-fly method from above is used, except with zero bias is on the gate, source, drain.

The pulsed on-the-fly NBTI measurement setup is implemented using a National Instruments chassis with a PXIE 6570 pulse driver and python control scripts. **Figure S6** characterizes the implementation of the controller timings and delays so that (1) we are aware of minimal NBTI time scales we can measure due to intrinsic controller/driver delays and (2) so our analysis properly includes these hidden delays.

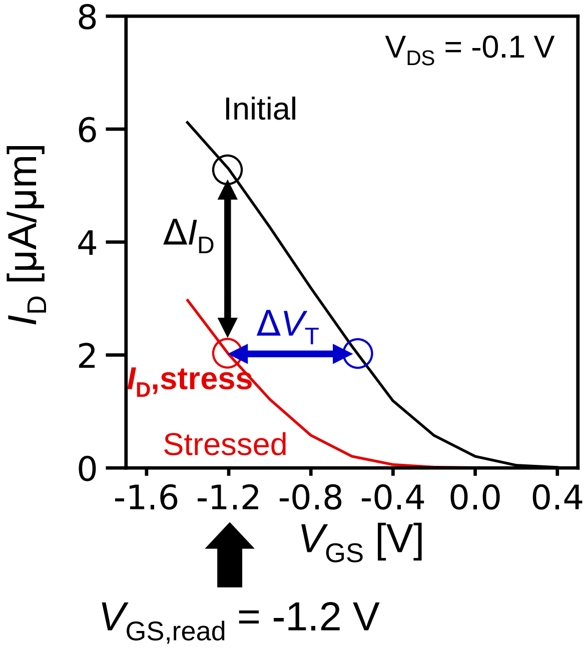

Figure S4. On-the-fly stressed V_T shift extraction. The black curve is an initial pulsed I_D-V_{GS} curve and the red curve is a pulsed I_D-V_{GS} curve after $V_{GS,stress}$ = -2 V for 1000 seconds followed by 1 second of post-stress relaxation.

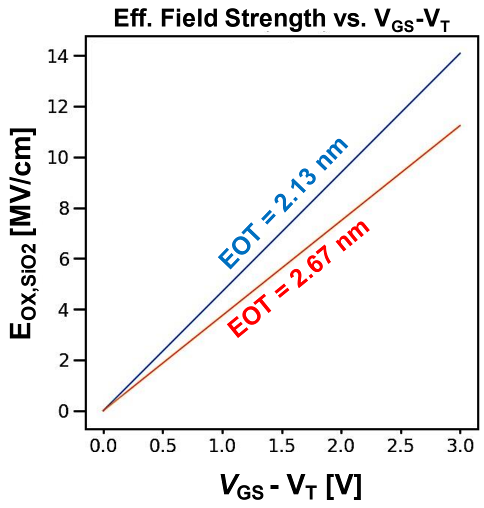

Figure S5. Effective Field Strength vs. Overdrive Voltage (V_{GS} – V_T). [25–27]

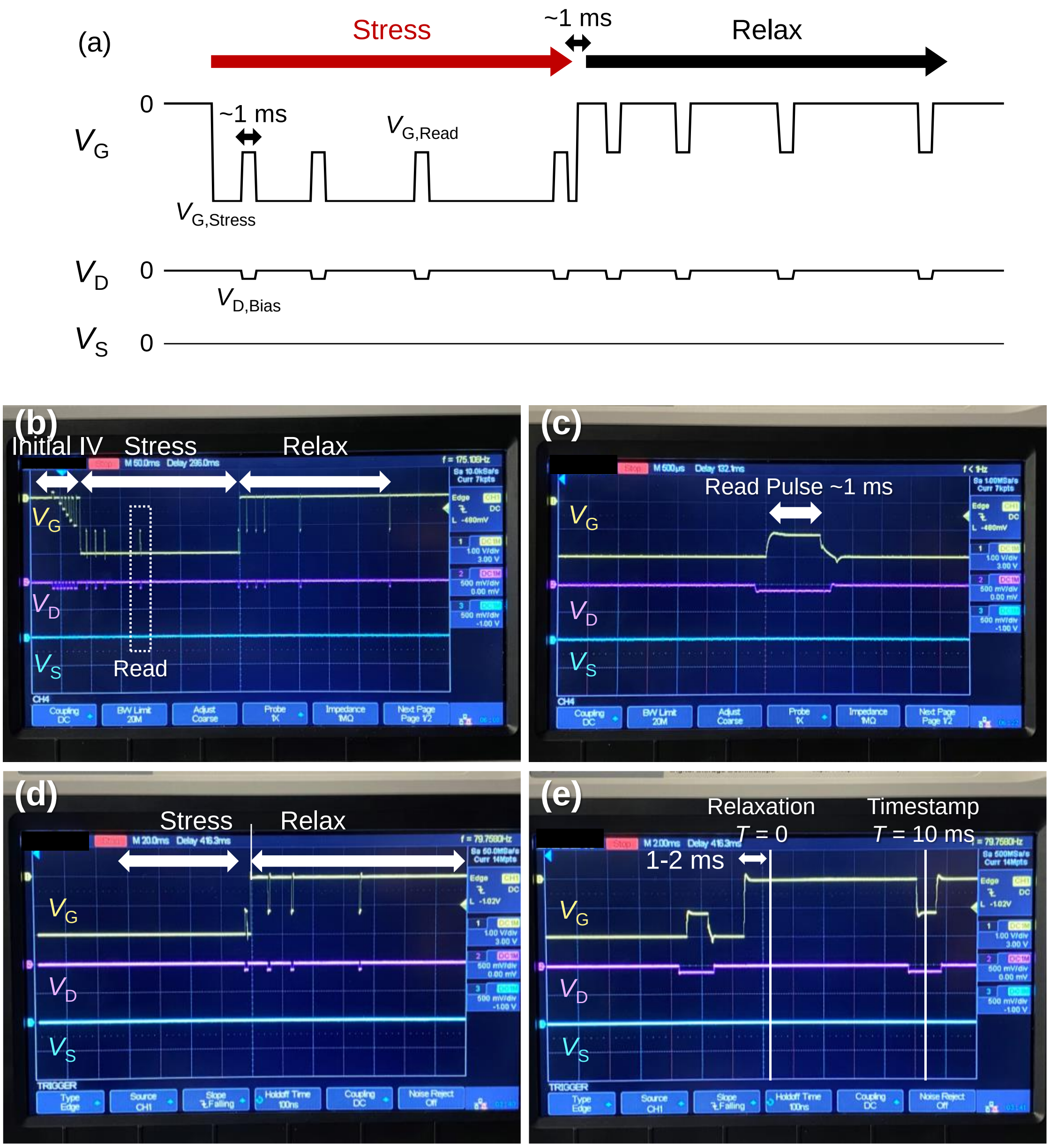

Figure S6. DC NBTI stress waveforms. (a) Diagram of typical DC NBTI stress and pulsed spot current measurement waveforms. Read measurements are spaced uniformly in log time. (b, c) Implementation verification demonstrating typical ~1 ms pulse widths (with range of 1 – 2 ms) and ~1–2 ms delay from stress to relaxation. (d) Stress and relax transition. (e) Zoom of delay from stress to relax.

S5. CNFET Relaxation Behavior

In silicon MOSFETs, Grasser *et al*. propose that NBTI follows a "universal relaxation" function [24,28],

$$z = t_r / T_{stress} \qquad \text{(S2)}$$

$$r = \frac{1}{1 + Bz^{\beta}} \qquad \text{(S3)}$$

where t_r is the relaxation time, T_{stress} is the final stress time, and $z = t_r/T_{stress}$ is termed the "normalized relaxation time." r is the relative ΔV_T relaxation fraction remaining = $\Delta V_T(t_r)/\Delta V_T(T_{stress})$ and B and β are empirical fitting parameters. This section determines if CNFETs follows this relaxation model.

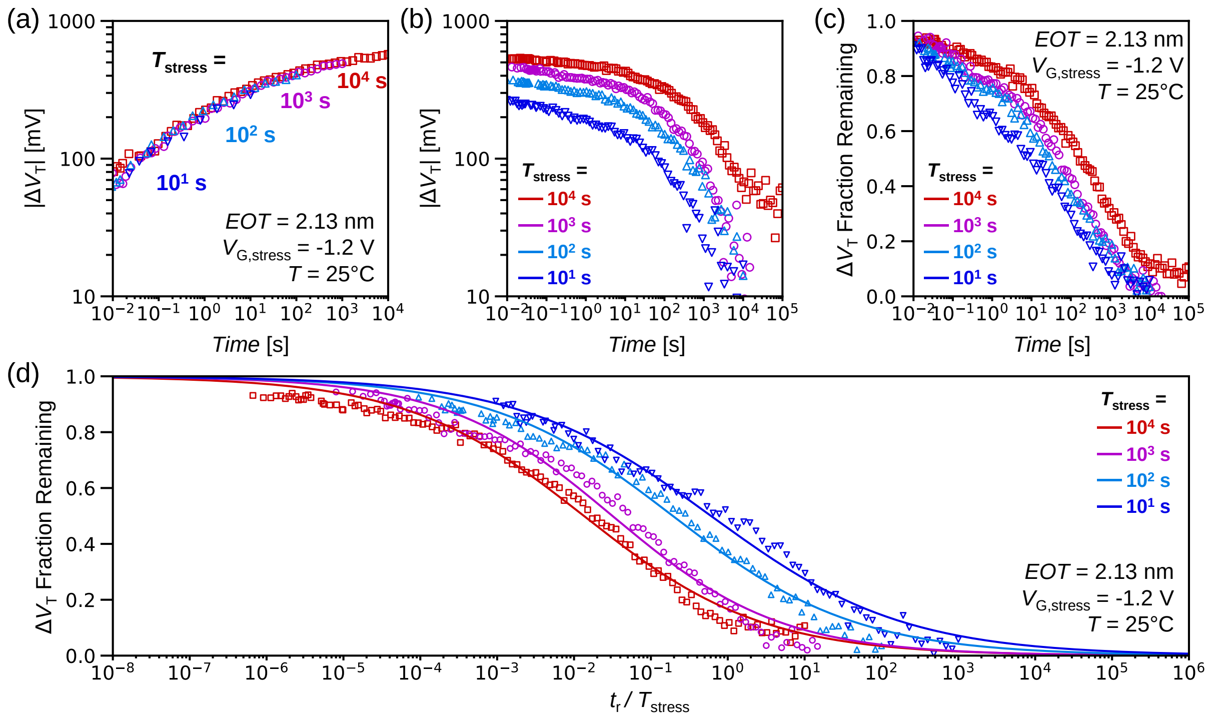

Figure S7. CNFET NBTI universal relaxation fitting. (a) DC NBTI threshold voltage shift ΔV_T after different total stress times T_{stress} applied to the same CNFET (after fully relaxing). (b) Absolute ΔV_T relaxation and (c) relative ΔV_T relaxation for different T_{stress} time applied in (a). (d) Universal relaxation equation fitting to relative relaxation versus time from (c). Measurements at room temperature, *EOT* ≈ 2.13 nm, stress voltage $V_{G,stress}$ = -1.2 V.

Figure S7 presents relaxation characterization for the same burned-in CNFET (1000 s at $V_{G,stress}$ = -2 V) at room temperature for several different stress times. The same CNFET is measured

during stress and relaxation, and then it is allowed to further relax such that the initial V_T recovers to within measurement noise. **Figure S7a** shows that all NBTI stress ΔV_T versus time traces overlap nearly identically, indicating the CNFET is adequately relaxed for each measurement. The order of stress/relaxation characterization steps was done as follows for total stress times: 10^4, 10^3, 10^2, and 10^1 s. While the universal relaxation function can fit the shape in the core of the relaxation, there are several notable differences in the time shift and the tails of the function (e.g. $z \ll 1$ and $z \gg 1$) relative to silicon:

1. Unexpected normalized relaxation time shift versus stress time: In silicon relaxation, by transforming to normalized relaxation time, the relative relaxation fraction is expected to occur at the same rate and all curves in **Figure S7d** are expected to overlap [28]. In CNFETs measured here, we observe that silicon "normalized relaxation time" does not apply to these measurements, as the relative relaxation vs normalized time curves do not overlap. This observation also does not follow prior reported graphene and MoS_2 relaxation which follows universal relaxation equation [29,30].
2. Initial relaxation is slow then accelerates: Silicon NBTI relative relaxation typically follows a roughly linear initial relaxation with an exponential slower tail [11,24,28]. The opposite is observed here (**Figure S7c**). The initial CNFET relaxation begins slower, then accelerates. For the $T_{stress} = 10^2$, 10^3, 10^4 second relaxation curves in **Figure S7c**, this corner can be seen at $t_r \sim 10$ seconds.
3. Longer term relaxation does not slow down: Silicon NBTI at longer relaxation time scale typically shows an exponentially slower recovery tail [11,24,28]. In the measured CNFET relaxation, other than the $T_{stress} = 10^4$ second curve which shows this behavior, all other curves shown in **Figure S7b,c** do not have a slower tail and continue to relax at the same rate. The ultimate relaxation ΔV_T recovery is limited by equipment noise floor and unintentional NBTI induced from the pulsed drain current measurement itself ($V_{G,read} = -0.6$ V). It is notable that even with this continued unintentional NBTI from the read sampling gate bias, the relaxation continues at the same rate without an exponentially slower tail.

The CNFET relaxation dynamics observed are significantly different than silicon NBTI and prior reported work on graphene and MoS_2. Significant future work is required to explore the physical origin for the CNFET relaxation dynamics observed here and why previous universal relaxation

dynamics do not apply here. Additionally, future work is required to study CNFET relaxation dynamics at different temperatures.

S6. CNFET AC/Pulsed NBTI Behavior

For many circuit applications, the CNFET will not be held at a constant DC bias during the operation. Rather, the CNFET gate, source, and drain will be pulsed on and off. This allows time for the CNFET to relax between stress ΔV_T, which reduces the long-term buildup of NBTI ΔV_T. This is modeled and characterized by AC NBTI stress performed at frequency f with duty cycle $D = t_{stress}/(t_{stress} + t_{relax})$ which is the fraction of each cycle that the stress voltage is applied. AC stress has the added complexity of sampling after the stress or relaxation part of the AC waveform, which may change the frequency and duty cycle dependence of the measured NBTI. As discussed by Tsai et al., taking samples after stress will capture fast traps while taking samples after relaxation portion can allow the fast traps to relax such that mainly the deeper trap component is captured [31]. **Figure S8** shows the different sampling modes and measured implementation are depicted. The notation used here is “Mode RS” for relax then stress then measure and “Mode SR” for stress then relax then measure. The AC waveform is a square pulsed wave and the duty cycle refers to the fraction of the AC period when the stress voltage is applied. Our AC stress experimental setup AC frequency is limited to a max of ~10 MHz for a duty cycling range of 0.1 to 0.9 (due to minimum controller pulse time scale of 10 ns plus some timing margin for accuracy). The typical read pulse time in AC stress is 2 ms. We use the same initial pulsed I_D-V_{GS} sweep method and same $V_{D,read}$ drain bias as in DC. Relaxation measurement is unchanged with AC stress. Note 10 MHz is max controller frequency, limited by minimum 10 ns driver resolution. At 10 MHz, the RS and SR pattern implementations result in different waveforms due to driver limitations, and the duty cycle is constrained between 0.2 and 0.8. Lower frequencies are not affected by this and RS and SR patterns are identical.

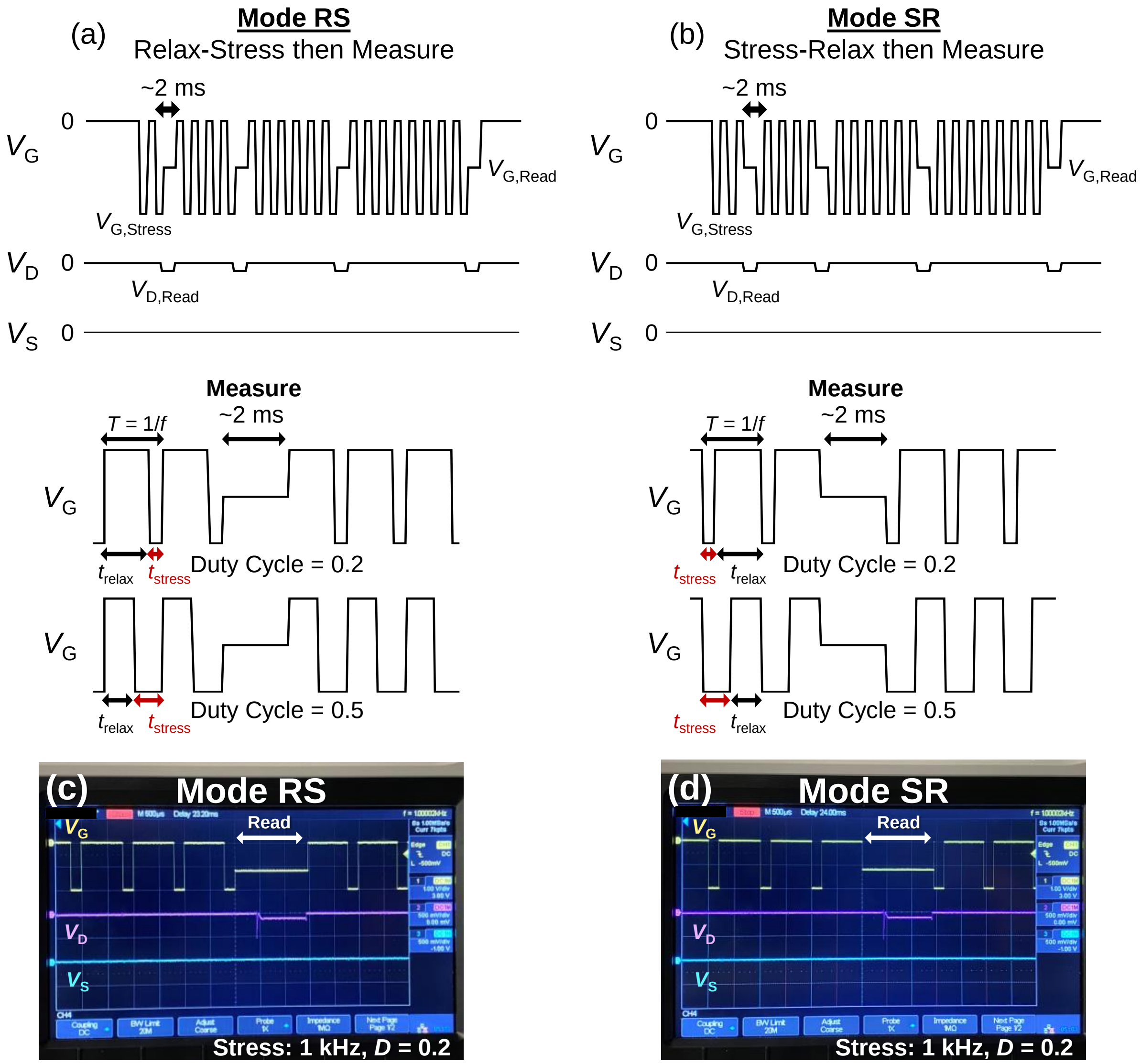

Figure S8. AC NBTI stress modes. Diagram of AC NBTI pulsed stress and pulsed measurement waveforms in (a) "Mode RS" (relax then stress then measure) and (b) "Mode SR" (stress then relax then measure). (c) "RS" and (d) "SR" waveform snapshots from oscilloscope.

Figure S9 shows room temperature AC NBTI threshold shift ΔV_T measured for a range of operating frequency (1 kHz to 1 MHz) under Mode RS and Mode SR stress at duty cycle $D = 50\%$. All measurements are performed on the same burned-in CNFET and it is relaxed between measurements such that initial V_T is the same (within measurement noise). The time axis is "cumulative stress time" which only sums the portions of time during the AC stress waveform that the CNFET is actually stressed, *i.e.* relaxation time is not included, so that the comparison is fair between DC stress and different AC stress frequencies and duty cycles. There is a consistent ~50%

reduction in NBTI ΔV_T reduction after 1000 seconds stress time, across the characterized frequency range 1 kHz to 1 MHz.

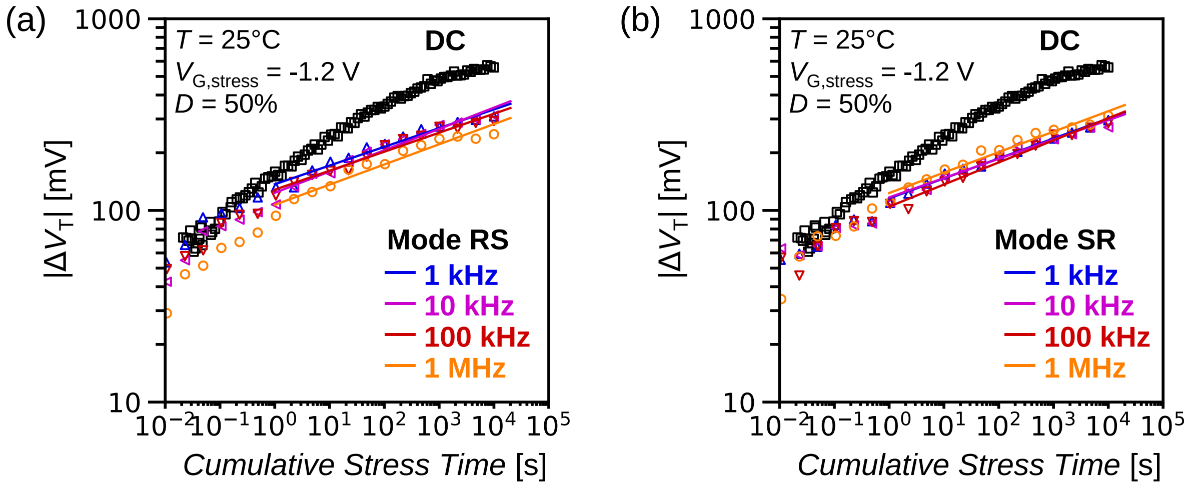

Figure S9. AC NBTI versus frequency at room temperature. AC NBTI ΔV_T shift for $EOT \approx$ 2.13 nm at $V_{G,stress}$ = -1.2 V across a range of frequency using stress waveforms (a) Mode RS (relax-stress-measure) and (b) Mode SR (stress-relax-measure). Controller ΔV_T read latency 1-2 ms.

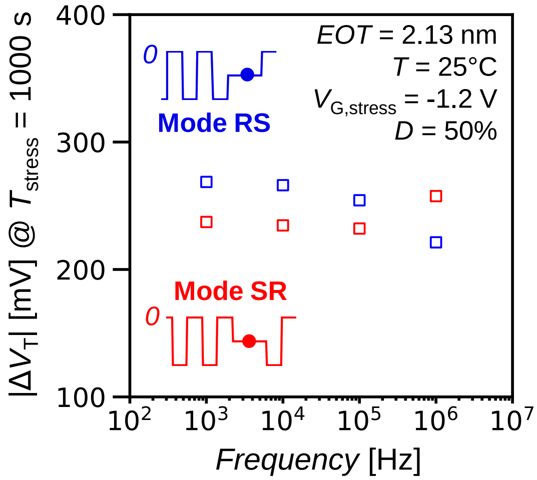

Figure S10. AC NBTI threshold shift vs frequency after fixed stress time. AC NBTI versus frequency sampled at T_{stress} = 1000 seconds from Figure S5. Both Mode RS and Mode SR show no clear frequency dependence. And there is no clear difference between Mode RS and Mode SR.

Figure S10 plots the sampled ΔV_T at T_{stress} = 1000 seconds, showing no frequency dependence and no difference between Mode RS and Mode SR measurements. This is unlike silicon which sees reduction in ΔV_T versus frequency for Mode RS but no frequency dependence for Mode SR [31]. The

physical expectation is that Mode RS should capture ΔV_T from both "deep" and "shallow" traps, while Mode SR discharges shallow traps whose time constants are less than the relaxation portion of the measurement pattern so that only ΔV_T from deep traps is captured [31]. Possible explanations are that (1) the time scale of discharging is significantly longer than the relaxation portion at all frequencies characterized or (2) the shallow trap discharging is too fast for our measurement equipment to capture (due to 1-2 ms read latency). Future work using measurement equipment with shorter read sampling latency or manually probing NBTI relaxation using an oscilloscope will be required to understand the relaxation dynamics at time scales <1 μs.

S7. Additional AC NBTI Duty Cycle Characterization

Reducing AC stress duty cycle D is expected to reduce accumulated NBTI ΔV_T. **Figure S11** shows room temperature AC NBTI ΔV_T measured with 1 MHz Mode RS and Mode SR stress across a range of duty cycles D. The AC measurements were performed on the same CNFET that is sufficiently relaxed between measurements to the same initial V_T (within measurement noise), but the DC measurement is taken from a separate CNFET with similar V_T. The observed trend is reduction in ΔV_T with lower duty cycle at stress times $>10^2$ seconds. **Figure S12** shows the plotted trend in ΔV_T for Mode RS and Mode SR measurements sampled at T_{stress} = 10000 seconds, alongside a model fit using a silicon AC NBTI model based on interface trap tunneling, originally developed by Tewksbury (1992) [6,31],

$$\Delta V_T(t_s, t_r) = \frac{qD_{ot}x_o}{C_{ox}}(E_F - E_{F0})\log\left(1 + \frac{\tau_{oe}t_{stress}}{\tau_{oc}t_{relax}}\right) = A\log\left(1 + B\frac{t_{stress}}{t_{relax}}\right) \tag{S4}$$

$$\frac{t_{stress}}{t_{relax}} = \frac{D}{1-D} \tag{S5}$$

where D_{ot} is the trap density in the oxide, x_o is a characteristic tunneling depth, C_{ox} is the gate dielectric capacitance, $E_F - E_{F0}$ is the energy range of traps, τ_{oc} and τ_{oe} are the trap capture and emission time constants, and t_{stress} and t_{relax} are the stress and relax time portions of the periodic AC stress waveform. For model fitting, the trap dynamics are simplified into two empirical fitting parameters A and B. The general shape of this model fits the measured duty cycle dependence in silicon undergoing AC NBTI stress. However, the model may need modifications to account for

trap spatial distribution in the gate oxide (*i.e.* D_{ot}(x) spatial distribution in the oxide) [31,32]. Our fit to this model (solid line in **Figure S12**) shows that room temperature AC NBTI in CNFET also generally follows this same model. But unlike in silicon [31], we see no observed difference between Mode RS and Mode SR trends or fit (both fits are using the same parameters).

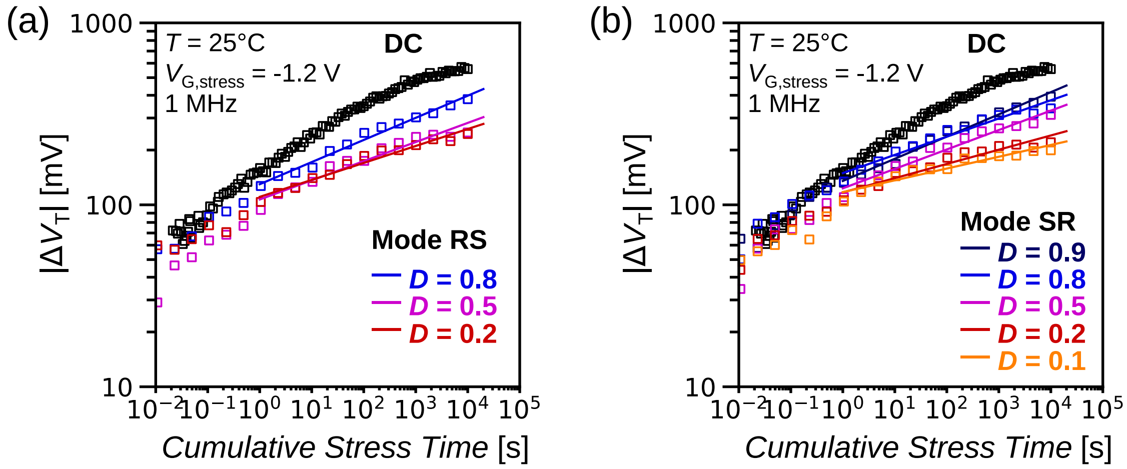

Figure S11. AC NBTI versus duty cycle at room temperature. AC NBTI ΔV_T shift for *EOT* ≈ 2.13 nm, $V_{G,stress}$ = -1.2 V, AC frequency 1 MHz, across a range of duty cycles *D*, using stress waveforms (a) Mode RS (relax-stress-measure) and (b) Mode SR (stress-relax-measure). Controller ΔV_T read latency 1-2 ms.

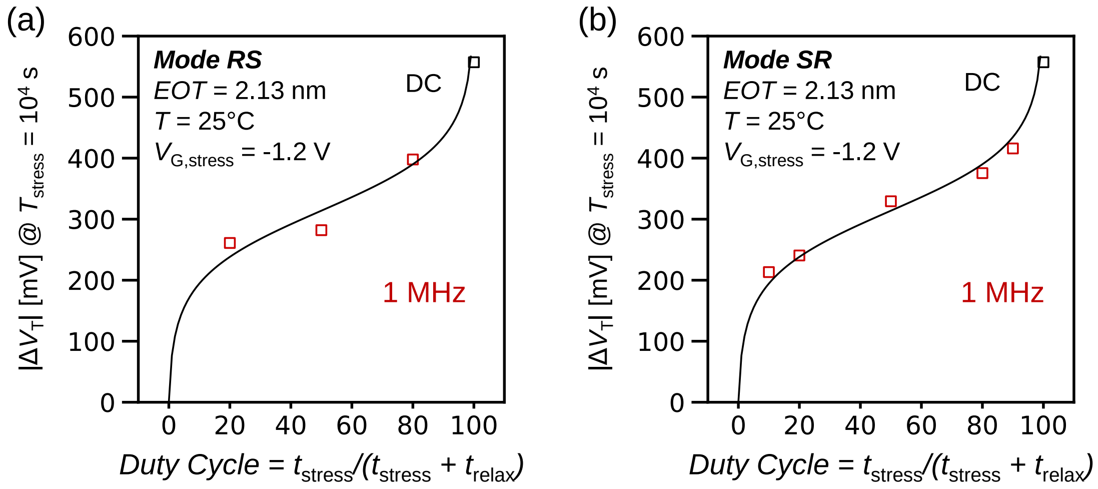

Figure S12. AC NBTI duty cycle model at room temperature. Red scatter points are AC NBTI ΔV_T shift after $V_{G,stress}$ = -1.2 V applied for 10^4 s cumulative stress time at 1 MHz versus duty cycle, sampled using (a) Mode RS (relax-stress-measure) and (b) Mode SR (stress-relax-measure). AC NBTI measurements were performed on the same CNFET (*EOT* ≈ 2.13 nm) with >18000 s of relaxation between measurements. DC NBTI is on a separate CNFET with similar V_T, I_D-V_{GS}, and NBTI characteristics. Solid line is a model fit using Equation 3.

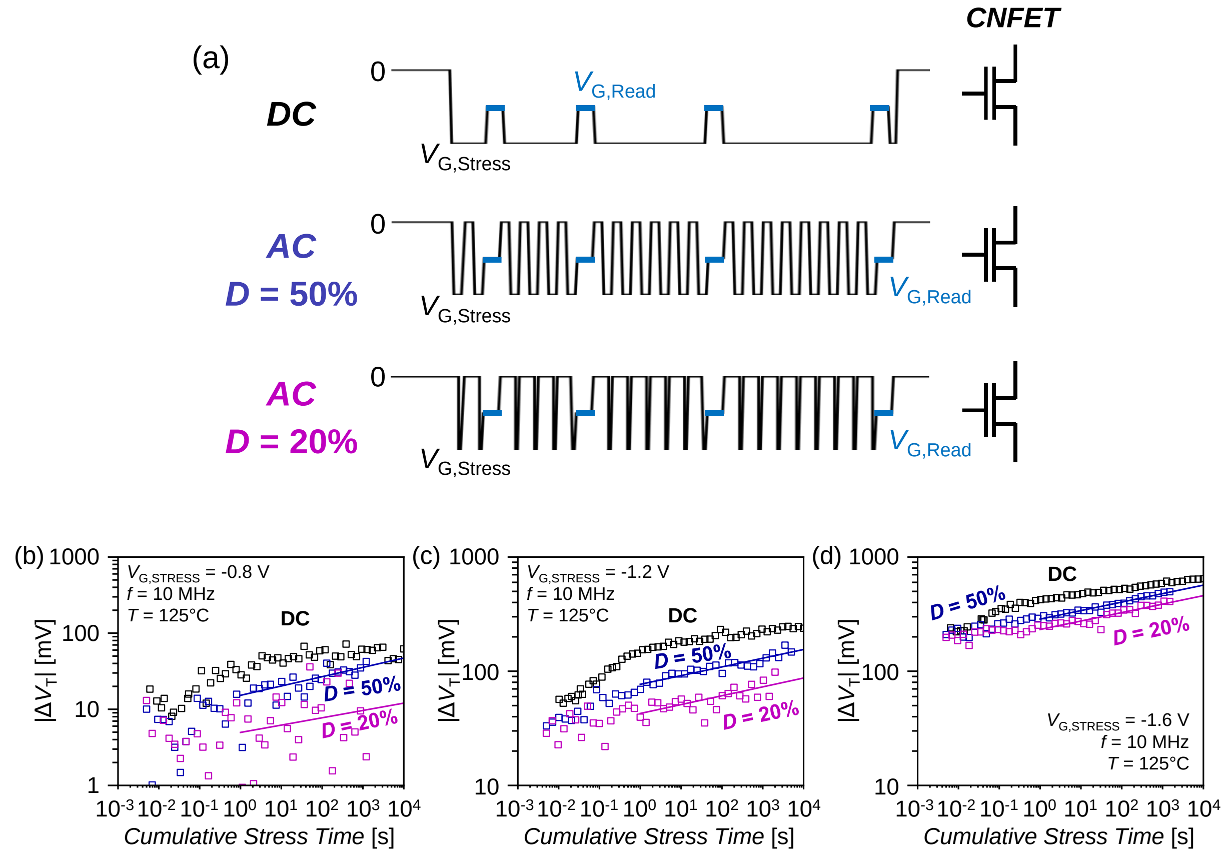

Figure S13. AC NBTI at 125°C at different duty cycle and stress bias. (a) Mode RS stress waveforms at 10 MHz. CNFETs have *EOT* ≈ 2.13 nm. AC NBTI ΔV_T versus time at different duty cycles *D* for gate biases (b) $V_{G,stress}$ = -0.8 V, (c) $V_{G,stress}$ = -1.2 V, (d) $V_{G,stress}$ = -1.6 V.

Figure S13 shows similar reductions in ΔV_T under AC NBTI with lower duty cycle at elevated temperature 125°C at 10 MHz using Mode RS stress, across a range of gate stress bias $V_{G,stress}$.

S8. AC NBTI Time-to-Failure Improvement

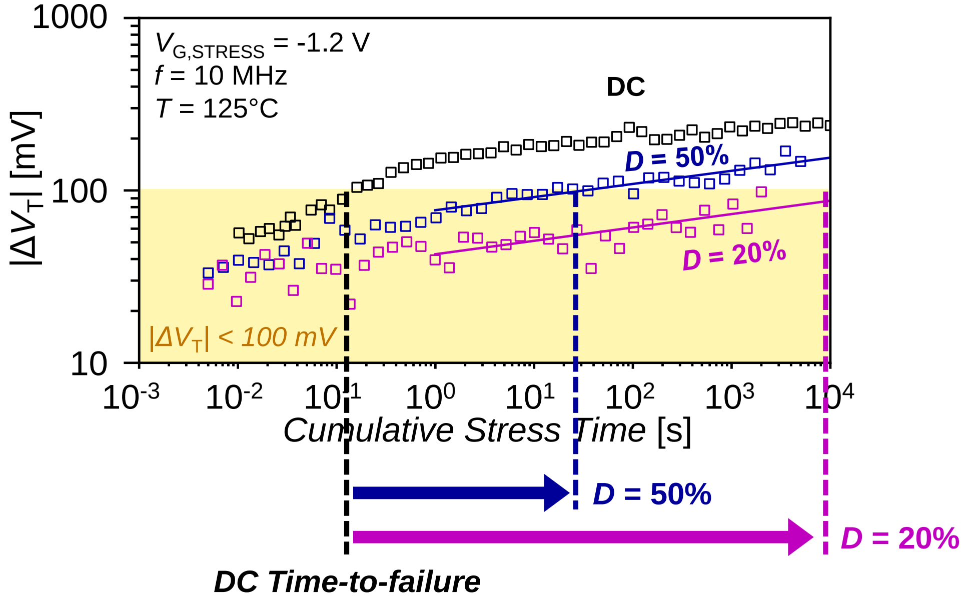

Figure S14. AC NBTI time-to-failure enhancement example versus duty cycle model at 125°C and 10 MHz. For an example NBTI tolerance of $|\Delta V_T| < 100$ mV before "failure", the CNFET DC NBTI time-to-failure when this limit is reached is the dashed vertical black line. By using duty cycled AC stress, with D = 50% this time-to-failure is extended by $>10^2\times$, and with D = 20% this time-to-failure is extended by $>10^4\times$. Mode RS stress at 10 MHz with $V_{G,stress}$ = -1.2 V, same measurements as in **Figure S13**.

S9. I_D-V_{GS} Measurement Hysteresis Example

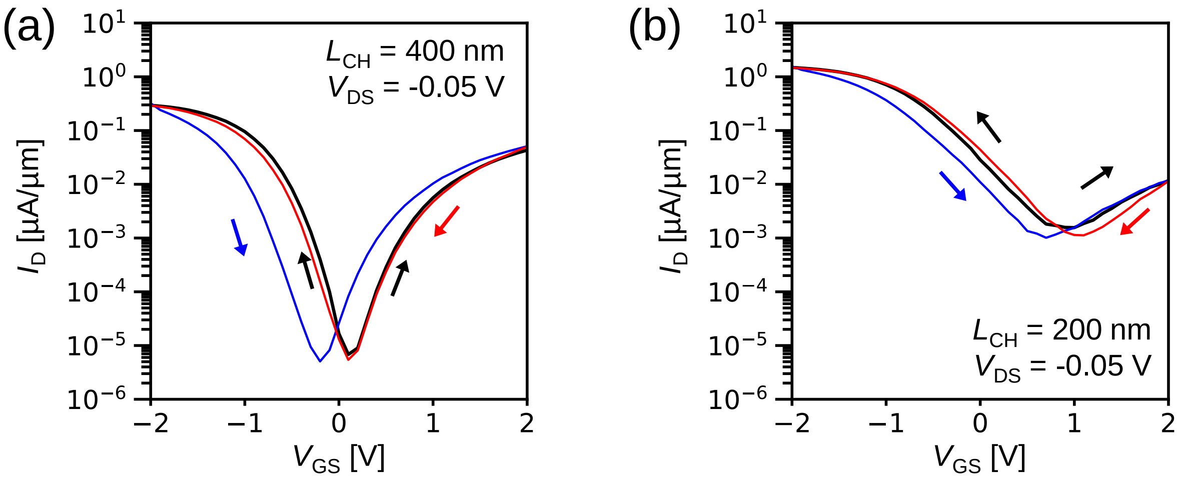

Figure S15. Measurement Direction Hysteresis: I_D-V_{GS} sweeps with different sweep directions: (blue) -2 V to 2 V; (red) 2 V to -2 V; (black) 0 to 2 V, relax 10^4s, and 0 to -2 V (black). These are measured for channel lengths (a) L_{CH} = 400 nm and (b) L_{CH} = 200 nm.

S10. C-V Characterized from Metal-Insulator-Metal Capacitors on Wafers

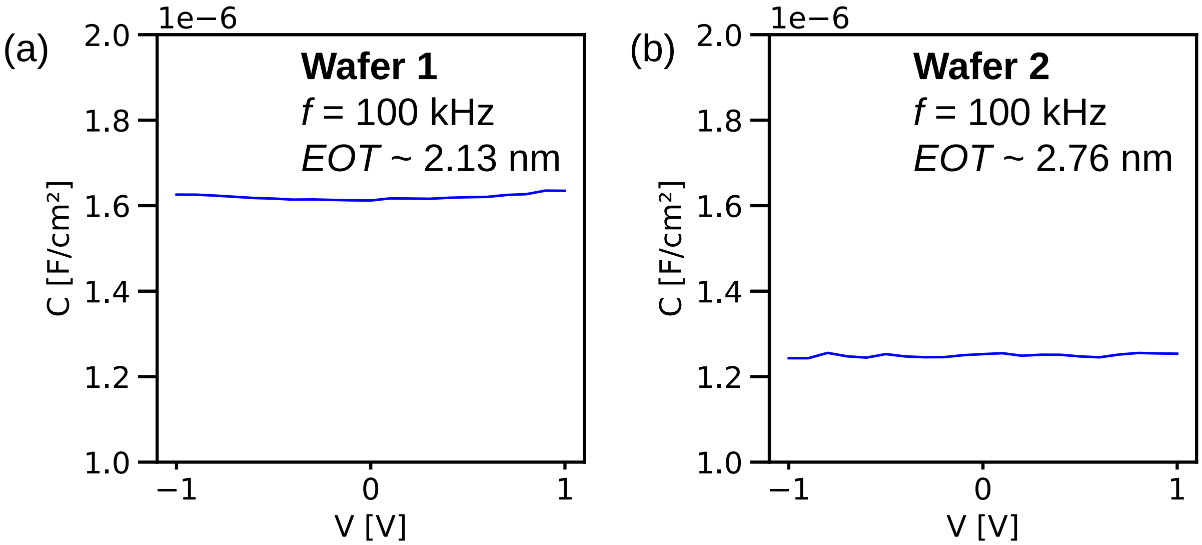

Figure S16. C-V measured from metal-insulator-metal capacitor test structures on the same wafer next to the FETs characterized for BTI in the manuscript, used to characterize gate dielectric capacitance and (a) *EOT* = 2.13 nm and (b) *EOT* = 2.76 nm. Insulator is the gate dielectric. C-V measured at frequency *f* = 100 kHz. These are the same wafers as in **Figure S3**.

REFERENCES

(1) Kim, W.; Javey, A.; Vermesh, O.; Wang, Q.; Li, Y.; Dai, H. Hysteresis Caused by Water Molecules in Carbon Nanotube Field-Effect Transistors. *Nano Lett.* **2003**, *3* (2), 193–198. https://doi.org/10.1021/nl0259232.

(2) Estrada, D.; Dutta, S.; Liao, A.; Pop, E. Reduction of Hysteresis for Carbon Nanotube Mobility Measurements Using Pulsed Characterization. *Nanotechnology* **2010**, *21* (8), 085702. https://doi.org/10.1088/0957-4484/21/8/085702.

(3) Park, R. S.; Hills, G.; Sohn, J.; Mitra, S.; Shulaker, M. M.; Wong, H.-S. P. Hysteresis-Free Carbon Nanotube Field-Effect Transistors. *ACS Nano* **2017**, *11* (5), 4785–4791. https://doi.org/10.1021/acsnano.7b01164.

(4) Lau, C. A Manufacturing Methodology for Carbon Nanotube-Based Digital Systems: From Devices, to Doping, to System Demonstrations, Massachusetts Institute of Technology, 2022. https://dspace.mit.edu/handle/1721.1/143373.

(5) Schroder, D. K. Negative Bias Temperature Instability: What Do We Understand? *Microelectronics Reliability* **2007**, *47* (6), 841–852. https://doi.org/10.1016/j.microrel.2006.10.006.

(6) Tewksbury, T. L. Relaxation Effects in MOS Devices Due to Tunnel Exchange with Near-Interface Oxide Traps, Massachusetts Institute of Technology, 1992. https://dspace.mit.edu/handle/1721.1/13238.

(7) Schroder, D. K. Negative Bias Temperature Instability (NBTI): Physics, Materials, Process and Circuit Issues, 2005. https://ewh.ieee.org/r5/denver/sscs/2005_08_Schroder.html.

(8) Young, M. J.; Bedford, N. M.; Yanguas-Gil, A.; Letourneau, S.; Coile, M.; Mandia, D. J.; Aoun, B.; Cavanagh, A. S.; George, S. M.; Elam, J. W. Probing the Atomic-Scale Structure of Amorphous Aluminum Oxide Grown by Atomic Layer Deposition. *ACS Appl. Mater. Interfaces* **2020**, *12* (20), 22804–22814. https://doi.org/10.1021/acsami.0c01905.

(9) Sah Chih-Tang. Evolution of the MOS Transistor-from Conception to VLSI. *Proc. IEEE* **1988**, *76* (10), 1280–1326. https://doi.org/10.1109/5.16328.

(10) Castro, P. L.; Deal, B. E. Low-Temperature Reduction of Fast Surface States Associated with Thermally Oxidized Silicon. *J. Electrochem. Soc.* **1971**, *118* (2), 280. https://doi.org/10.1149/1.2408016.

(11) Rangan, S.; Mielke, N.; Yeh, E. C. C. Universal Recovery Behavior of Negative Bias Temperature Instability. In *IEEE International Electron Devices Meeting 2003*; IEEE: Washington, DC, USA, 2003; p 14.3.1-14.3.4. https://doi.org/10.1109/IEDM.2003.1269294.

(12) Grasser, T.; Aichinger, Th.; Pobegen, G.; Reisinger, H.; Wagner, P.-J.; Franco, J.; Nelhiebel, M.; Kaczer, B. The “Permanent” Component of NBTI: Composition and Annealing. In *2011 International Reliability Physics Symposium*; IEEE: Monterey, CA, USA, 2011; p 6A.2.1-6A.2.9. https://doi.org/10.1109/IRPS.2011.5784543.

(13) Grasser, T.; Waltl, M.; Wimmer, Y.; Goes, W.; Kosik, R.; Rzepa, G.; Reisinger, H.; Pobegen, G.; El-Sayed, A.; Shluger, A.; Kaczer, B. Gate-Sided Hydrogen Release as the Origin of “Permanent” NBTI Degradation: From Single Defects to Lifetimes. In *2015 IEEE International Electron Devices Meeting (IEDM)*; IEEE: Washington, DC, USA, 2015; p 20.1.1-20.1.4. https://doi.org/10.1109/IEDM.2015.7409739.

(14) Grasser, T.; Kaczer, B.; Goes, W.; Reisinger, H.; Aichinger, T.; Hehenberger, P.; Wagner, P.-J.; Schanovsky, F.; Franco, J.; Toledano Luque, M.; Nelhiebel, M. The Paradigm Shift in

Understanding the Bias Temperature Instability: From Reaction–Diffusion to Switching Oxide Traps. *IEEE Trans. Electron Devices* **2011**, *58* (11), 3652–3666. https://doi.org/10.1109/TED.2011.2164543.
(15) Gallington, L.; Ghadar, Y.; Skinner, L.; Weber, J.; Ushakov, S.; Navrotsky, A.; Vazquez-Mayagoitia, A.; Neuefeind, J.; Stan, M.; Low, J.; Benmore, C. The Structure of Liquid and Amorphous Hafnia. *Materials* **2017**, *10* (11), 1290. https://doi.org/10.3390/ma10111290.
(16) Hsu, A.; Wang, H.; Kim, K. K.; Kong, J.; Palacios, T. Impact of Graphene Interface Quality on Contact Resistance and RF Device Performance. *IEEE Electron Device Lett.* **2011**, *32* (8), 1008–1010. https://doi.org/10.1109/LED.2011.2155024.
(17) Srimani, T.; Ding, J.; Yu, A.; Kanhaiya, P.; Lau, C.; Ho, R.; Humes, J.; Kingston, C. T.; Malenfant, P. R. L.; Shulaker, M. M. Comprehensive Study on High Purity Semiconducting Carbon Nanotube Extraction. *Adv Elect Materials* **2022**, *8* (9), 2101377. https://doi.org/10.1002/aelm.202101377.
(18) Schroder, D. K. *Semiconductor Material and Device Characterization*, 1st ed.; Wiley, 2005. https://doi.org/10.1002/0471749095.
(19) Park, R. S.; Shulaker, M. M.; Hills, G.; Suriyasena Liyanage, L.; Lee, S.; Tang, A.; Mitra, S.; Wong, H.-S. P. Hysteresis in Carbon Nanotube Transistors: Measurement and Analysis of Trap Density, Energy Level, and Spatial Distribution. *ACS Nano* **2016**, *10* (4), 4599–4608. https://doi.org/10.1021/acsnano.6b00792.
(20) Li, S.; Chao, T.-A.; Gilardi, C.; Safron, N.; Su, S.-K.; Zeevi, G.; Bechdolt, A. D.; Passlack, M.; Oberoi, A.; Lin, Q.; Zhang, Z.; Wang, K.; Kashyap, H.; Liew, S.-L.; Hou, V. D.-H.; Kummel, A.; Radu, L.; Pitner, G.; Wong, H.-S. P.; Mitra, S. High-Performance and Low Parasitic Capacitance CNT MOSFET: 1.2 mA/Mm at V_{DS} of 0.75 V by Self-Aligned Doping in Sub-20 Nm Spacer. In *2023 International Electron Devices Meeting (IEDM)*; IEEE: San Francisco, CA, USA, 2023; pp 1–4. https://doi.org/10.1109/IEDM45741.2023.10413827.
(21) McClellan, C. J.; Yalon, E.; Smithe, K. K. H.; Suryavanshi, S. V.; Pop, E. High Current Density in Monolayer MoS_2 Doped by AlO_x. *ACS Nano* **2021**, *15* (1), 1587–1596. https://doi.org/10.1021/acsnano.0c09078.
(22) Zhao, P.; Azcatl, A.; Bolshakov, P.; Moon, J.; Hinkle, C. L.; Hurley, P. K.; Wallace, R. M.; Young, C. D. Effects of Annealing on Top-Gated MoS2 Transistors with HfO2 Dielectric. *Journal of Vacuum Science & Technology B, Nanotechnology and Microelectronics: Materials, Processing, Measurement, and Phenomena* **2017**, *35* (1), 01A118. https://doi.org/10.1116/1.4974220.
(23) Zhao, P.; Padovani, A.; Bolshakov, P.; Khosravi, A.; Larcher, L.; Hurley, P. K.; Hinkle, C. L.; Wallace, R. M.; Young, C. D. Understanding the Impact of Annealing on Interface and Border Traps in the Cr/HfO_2/Al_2O_3/MoS_2 System. *ACS Appl. Electron. Mater.* **2019**, *1* (8), 1372–1377. https://doi.org/10.1021/acsaelm.8b00103.
(24) Kaczer, B.; Grasser, T.; Roussel, J.; Martin-Martinez, J.; O'Connor, R.; O'Sullivan, B. J.; Groeseneken, G. Ubiquitous Relaxation in BTI Stressing—New Evaluation and Insights. In *2008 IEEE International Reliability Physics Symposium*; IEEE: Phoenix, AZ, 2008; pp 20–27. https://doi.org/10.1109/RELPHY.2008.4558858.
(25) Cho, M.; Lee, J.-D.; Aoulaiche, M.; Kaczer, B.; Roussel, P.; Kauerauf, T.; Degraeve, R.; Franco, J.; Ragnarsson, L.-Å.; Groeseneken, G. Insight Into N/PBTI Mechanisms in Sub-1-Nm-EOT Devices. *IEEE Trans. Electron Devices* **2012**, *59* (8), 2042–2048. https://doi.org/10.1109/TED.2012.2199496.

(26) Mukhopadhyay, S.; Joshi, K.; Chaudhary, V.; Goel, N.; De, S.; Pandey, R. K.; Murali, K. V. R. M.; Mahapatra, S. Trap Generation in IL and HK Layers during BTI / TDDB Stress in Scaled HKMG N and P MOSFETs. In *2014 IEEE International Reliability Physics Symposium*; IEEE: Waikoloa, HI, 2014; p GD.3.1-GD.3.11. https://doi.org/10.1109/IRPS.2014.6861146.
(27) Degraeve, R.; Aoulaiche, M.; Kaczer, B.; Roussel, Ph.; Kauerauf, T.; Sahhaf, S.; Groeseneken, G. Review of Reliability Issues in High-k/Metal Gate Stacks. In *2008 15th International Symposium on the Physical and Failure Analysis of Integrated Circuits*; IEEE: singapore, 2008; pp 1–6. https://doi.org/10.1109/IPFA.2008.4588195.
(28) Grasser, T.; Gös, W.; Sverdlov, V.; Kaczer, B. The Universality of NBTI Relaxation and Its Implications for Modeling and Characterization. In *2007 IEEE International Reliability Physics Symposium Proceedings. 45th Annual*; IEEE: Phoenix, AZ, USA, 2007; pp 268–280. https://doi.org/10.1109/RELPHY.2007.369904.
(29) Illarionov, Yu. Yu.; Smith, A. D.; Vaziri, S.; Ostling, M.; Mueller, T.; Lemme, M. C.; Grasser, T. Bias-Temperature Instability in Single-Layer Graphene Field-Effect Transistors. *Applied Physics Letters* **2014**, *105* (14), 143507. https://doi.org/10.1063/1.4897344.
(30) Illarionov, Yu. Yu.; Waltl, M.; Furchi, M. M.; Mueller, T.; Grasser, T. Reliability of Single-Layer MoS2 Field-Effect Transistors with SiO2 and hBN Gate Insulators. In *2016 IEEE International Reliability Physics Symposium (IRPS)*; IEEE: Pasadena, CA, USA, 2016; pp 5A-1-1-5A-1–6. https://doi.org/10.1109/IRPS.2016.7574543.
(31) Tsai, Y. S.; Jha, N. K.; Lee, Y.-H.; Ranjan, R.; Wang, W.; Shih, J. R.; Chen, M. J.; Lee, J. H.; Wu, K. Prediction of NBTI Degradation for Circuit under AC Operation. In *2010 IEEE International Reliability Physics Symposium*; IEEE: Garden Grove (Anaheim), CA, USA, 2010; pp 665–669. https://doi.org/10.1109/IRPS.2010.5488752.
(32) Huard, V.; Parthasarathy, C.; Rallet, N.; Guerin, C.; Mammase, M.; Barge, D.; Ouvrard, C. New Characterization and Modeling Approach for NBTI Degradation from Transistor to Product Level. In *2007 IEEE International Electron Devices Meeting*; IEEE: Washington, DC, USA, 2007; pp 797–800. https://doi.org/10.1109/IEDM.2007.4419068.
